# ATF2-driven osteogenic activity of enoxaparin sodium-loaded polymethylmethacrylate bone cement in femoral defect regeneration

**DOI:** 10.1186/s13018-023-04017-8

**Published:** 2023-08-31

**Authors:** Luobin Ding, Kangning Hao, Linchao Sang, Xiaoyu Shen, Ce Zhang, Dehao Fu, Xiangbei Qi

**Affiliations:** 1 Department of Orthopedic Surgery, The Third Hospital of Hebei Medical University, No. 139, Ziqiang Road, Shijiazhuang, 050051 Hebei People’s Republic of China; 2 Department of Orthopedic Surgery, Third Hospital of Shijiazhuang, Shijiazhuang, 050000 People’s Republic of China; 3 Department of Orthopedics, Shanghai General Hospital, Shanghai Jiao Tong University School of Medicine, Shanghai, 200000 People’s Republic of China

**Keywords:** Osteogenic differentiation, Bone marrow mesenchymal stem cells, Femoral defect regeneration, Femoral defect repair, Polymethylmethacrylate bone cement, Enoxaparin sodium, ATF2, miR-335-5p

## Abstract

**Background:**

Polymethylmethacrylate (PMMA) bone cement loaded with enoxaparin sodium (PMMA@ES) has been increasingly highlighted to affect the bone repair of bone defects, but the molecular mechanisms remain unclear. We addressed this issue by identifying possible molecular mechanisms of PMMA@ES involved in femoral defect regeneration based on bioinformatics analysis and network pharmacology analysis.

**Methods:**

The upregulated genes affecting the osteogenic differentiation of bone marrow mesenchymal stem cells (BMSCs) were selected through bioinformatics analysis, followed by intersection with the genes of ES-induced differentiation of BMSCs identified by network pharmacology analysis. PMMA@ES was constructed. Rat primary BMSCs were isolated and cultured in vitro in the proliferation medium (PM) and osteogenic medium (OM) to measure alkaline phosphatase (ALP) activity, mineralization of the extracellular matrix, and the expression of RUNX2 and OCN using gain- or loss-of-function experiments. A rat femoral bone defect model was constructed to detect the new bone formation in rats.

**Results:**

ATF2 may be a key gene in differentiating BMSCs into osteoblasts. In vitro cell assays showed that PMMA@ES promoted the osteogenic differentiation of BMSCs by increasing ALP activity, extracellular matrix mineralization, and RUNX2 and OCN expression in PM and OM. In addition, ATF2 activated the transcription of miR-335-5p to target ERK1/2 and downregulate the expression of ERK1/2. PMMA@ES induced femoral defect regeneration and the repair of femoral defects in rats by regulating the ATF2/miR-335-5p/ERK1/2 axis.

**Conclusion:**

The evidence provided by our study highlighted the ATF2-mediated mechanism of PMMA@ES in the facilitation of the osteogenic differentiation of BMSCs and femoral defect regeneration.

**Supplementary Information:**

The online version contains supplementary material available at 10.1186/s13018-023-04017-8.

## Background

Mesenchymal stem cells, also known as mesenchymal stromal cells (MSCs), are widely distributed in the body and essential for tissue homeostasis and regeneration by primarily acting on immune cells [1, 2]. Bone marrow-derived mesenchymal stem cells (BMSCs) with enhanced osteogenic differentiation capacity are recognized as one of the most promising treatments for femoral bone defects [3–6]. Therefore, studying the mechanisms of osteogenic differentiation of BMSCs is significant for repairing femoral bone defects.

ATF2 functions as an important transcription factor that can exert physiological effects on regulating cellular adaptation to response to stress, hypoxia, and DNA damage [7, 8]. In addition, ATF2 can modify histone acetylation [9] and affect ATM activation by regulating TIP60 [10]. ATF2 transcriptional activity and expression are associated with diverse diseases, such as Kashin-Beck disease [11], obesity [12], and liver cirrhosis [13]. Of particular note, ATF2 with JUK and P38 promotes the apoptotic process of chondrocytes in cartilage tissues [11]. In addition, ATF2 and p38MAPK act as key regulatory proteins to promote the differentiation process of osteoblasts [14].

ATF2 acts as a transcription factor in the nucleus to regulate the expression of various key genes, affecting resistance to apoptosis, cell growth and differentiation, and DNA damage repair [15–17]. In the cytoplasm, ATF2 binds to and disrupts the HK1-VDAC complex on the mitochondrial membrane, leading to mitochondrial leakage to promote cell death [18, 19]. miR-335-5p, as a non-transcriptional RNA in the cell to regulate other genes, promotes cell death to affect tumor initiation, progression, metastasis, and drug resistance by regulating gene expression [20, 21]. Moreover, it has also been demonstrated that ATF2 can affect bone growth and differentiation by regulating gene expression to enhance or inhibit chondrocyte proliferation and growth plate progression [22]. A recent study has reported that lncRNA NEAT1 with high enhancer activity can be activated by ATF2, which induces the differentiation of MSCs into osteoblasts by promoting mitochondrial function [23]. Regarding skeletal differentiation and growth, miR-335-5p affects the development of osteoarthritis by targeting HBP1 [24]. It is also interesting that miR-335-5p can promote bone regeneration [25].

A recent study has demonstrated that miR-335 can inhibit lipopolysaccharide-mediated extracellular regulated protein kinase (ERK)1/2 activation in experimental models of Parkinson's disease [26]. Furthermore, it has been elucidated that inhibition of ERK1/2 activation in mature osteoblasts promotes bone formation via the mTOR signaling pathway [27]. Accumulating evidence has suggested that fluctuations in ERK phosphorylation levels play a key role in osteoblast self-renewal [28]. Another study has also confirmed the inhibitory effects of miR-335-5p on ERK1/2 phosphorylation [29]. Therefore, we hypothesize that ATF2 may activate miR-335-5p to suppress the ERK1/2 phosphorylation in a femoral defect regeneration model, ultimately promoting MSC differentiation towards osteoblasts and subsequent osteogenic function of osteoblasts. Our study investigated the possible molecular mechanisms involved in the targeted differentiation of BMSCs induced by polymethylmethacrylate (PMMA) bone cement loaded with sodium enoxaparin (PMMA@ES) for femoral defect regeneration.

## Materials and methods

### Microarray-based gene expression profiling and network pharmacology analysis

The GSE9451 microarray data for osteogenic differentiation of BMSCs were obtained from the GEO database and were annotated with the platform annotation file GPL570 [HG-U133_Plus_2] Affymetrix Human Genome U133 Plus 2.0 Array. Differential analysis for sample genes was performed using the R language “limma” package with |log_2_ FC|> 2 and *p*-value < 0.05 as the screening criteria. Heat maps and volcano maps were created by introducing differentially expressed genes (DEGs) using the R language “pheatmap” package.

The target genes related to ES were retrieved from the CTD database with an inference score ≥ 30 as the screening criterion, and the intersection of target genes between the two databases was obtained using the Draw Venn Diagram tool. The candidate target genes were then subjected to PPI analysis, and their downstream regulatory pathways were predicted by STRING. The nodes and edges of the PPI network were analyzed using the Cytoscape 3.5.1 software, and the Degree was sorted to filter the core genes of the network.

### Rat BMSC culture in vitro

Rat BMSCs (R7500, ScienCell, San Diego, CA, USA) were incubated in a proliferation medium (PM) containing Dulbecco’s modified Eagle’s medium (DMEM) 11054020, Gibco, Grand Island, NY), 10% (v/v) fetal bovine serum (FBS; F8687, Shanghai Sigma-Aldrich Trading Co., Ltd., Shanghai, China), 100 U/mL penicillin G, and 100 mg/mL streptomycin (15140148, Gibco), which was incubated with 5% CO_2_ and 100% humidity at 37 °C.

To induce osteogenesis and observe the effects of different treatment methods on the osteogenic differentiation ability of BMSCs, BMSCs were seeded in six-well plates at a density of 2 × 10^4^ cells/well [30]. When reaching 80% confluence, the cells were cultured in osteogenic medium (OM) supplemented with 5 mM β-glycerophosphate (G9422, Sigma-Aldrich (Shanghai) Trading Co., Ltd.) and 50 µg/mL ascorbic acid (BP461, Shanghai Sigma-Aldrich Trading Co., Ltd.).

### In vitro experiment protocols

The oe-ATF2 and other plasmids were purchased from Shanghai Yaji Biotechnology Co. (Shanghai, China). BMSCs were inoculated in 6-well plates. Upon cell, confluence reached 60–70%, 10 µL Lipofectamine™ 2000 (11668500, Invitrogen, Carlsbad, CA) and 4 µg plasmid were diluted in 250 µL Opti-MEM (11058021, Thermo Fisher Scientific Inc., Waltham, MA), respectively, for 6 min. The two were gently mixed, allowed to stand for 20 min, added to the culture well, and then cultured in an incubator with 5% CO_2_ at 37 °C. After 6 h, the medium was replaced with a complete medium, and cells continued to culture for 48 h. Cells were collected for RNA and total protein extraction, and transfection efficiency was measured by reverse transcription-quantitative polymerase chain reaction (RT-qPCR) and Western blot analysis [31, 32].

Cells were transfected with ATF2 overexpression (oe-ATF2) plasmid, oe-ERK1/2, short hairpin RNA (shRNA, sh-) targeting ATF2-1 (sh-ATF2-1), sh-ATF2-2, miR-335-5p inhibitor, and corresponding negative control (NC) plasmids oe-NC, sh-NC, and inhibitor NC, alone or in combination. Invogen Tech designed the shATF2 sequences. Co., Ltd. (Beijing, China) [33]. The knockdown sequences are shown in Additional file 5: Table S1.

### Construction of PMMA@ES

PMMA was prepared with 12% polyvinylpyrrolidone K-30 and 20% citric acid. The cement paste was then mixed into two phases: the liquid and solid phases. For the PMMA@ES group, PMMA was soaked with 0.5% ES buffer for 24 h to allow complete uptake of ES into PMMA by the material [34, 35].

### Alkaline phosphatase (ALP) activity assay

The BMSCs were inoculated in 96-well cell culture plates at a density of 1 × 10^5^ cells/well, with 3 replicates per group. After induction with PM or OM for 7 days, the relevant indexes were measured according to the ALP assay kit (A059-3-1, Nanjing Jiancheng Institute of Biological Engineering, Nanjing, China). With p-nitrophenyl phosphate (pNPP) as substrate, ALP activity was measured at 405 nm. ALP in the cells was developed. pNPP turned yellow when ALP dephosphorylated p-nitrophenyl phosphate. Next, the data were then analyzed according to the absorbance measured at 520 nm [36].

### Alizarin red staining (ARS)

BMSCs were transferred into the 24-well plates at a density of 2 × 10^5^ per well, with 3 replicates per group. After induction with PM or OM for 7 days, ARS was performed according to the ARS Kit (A5533, Sigma), which was performed at 24–26 °C for approximately 10 min, followed by washing with PBS and observing the level of stromal mineralization under an inverted microscope. To detect the concentration of calcium deposition, the alizarin red dye in BMSCs could be extracted with 400 µL 10% (w/v) sodium cetyl pyridinium chloride solution in 10 mM sodium phosphate solution for 10 min. The optical density (OD) value could be measured on a UV–Vis spectrometer at 562 nm [36].

### ChIP assay

The ChIP kit (KT101-02, Sai Cheng Biotechnology Co., Ltd., Guangzhou, China) was used to detect the enrichment of ATF2 in the promoter region of the miR-335-5p gene. In brief, upon cell confluency reached 70–80%, cells were fixed in 4% formaldehyde at room temperature for 10 min to cross-link the DNA and protein in the cells. After the termination of fixation, the cell lysate was obtained. After cross-linking, cells were randomly sonicated to obtain DNA fragments of 300–1000 bp. Next, the cells were centrifuged at 6540 g at 4 °C to obtain the supernatant. Immunoprecipitation was performed with rabbit anti-ATF2 (ab131168, 1:250, Abcam, Cambridge, UK), an antibody specific to the target protein, and Protein G Dynabeads (Invitrogen). The endogenous DNA–protein complexes were precipitated using Protein Agarose/Sepharose; the supernatant was aspirated and discarded after brief centrifugation, the non-specific complexes were washed, uncrosslinked overnight at 65 °C and the DNA fragments were recovered by phenol/chloroform extraction and purified for qPCR of the miR-335-5p gene promoter fragment [37–39]. miR-335-5p gene promoter region-specific primer sequences are shown in Additional file 5: Table S2.

### Dual-luciferase reporter assay

The miR-335-5p was predicted through the online database Targetscan to predict the binding sites of miR-335-5p to ERK1/2, respectively. The 3'UTR fragment of the ERK1/2 gene was cloned and amplified, and the PCR product was cloned into a polyclonal site downstream of the Luciferase gene (luc2) of pmirGLO (E1330, Promega, Madison, WI), named pERK1/2-WT (UGUACA). Next, site-directed mutagenesis was conducted on the binding sites of miR-335-5p to target genes predicted by bioinformatics. pERK1/2-MUT (CACGAU) reporter vector was constructed. It was transduced into human embryonic kidney cells HEK293T (iCell-h237, Sebacon Biotechnology Co., Ltd., Shanghai, China) with miR-335-5p mimic and NC, respectively, according to the instructions of Lipofectamine 3000 reagent. The luciferase activity was measured using the Dual-Luciferase® Reporter Assay System (E1910, Promega) and recorded by GloMax 96 microtiter plate. Renilla luciferase was used as an internal reference [40, 41].

### RT-qPCR

Total RNA was extracted from cells by TRIzol (16096020, Invitrogen). For mRNA, reverse transcription was performed using a reverse transcription kit (04897030001, Roche, Basel, Switzerland) to obtain the corresponding cDNA. For miRNA, a PolyA tailing kit (B532451, Shanghai Sangon Biotechnology Co. Ltd., Shanghai, China) was used to obtain the cDNA containing PolyA-tailed miRNA. The reverse transcribed cDNA was diluted to 50 ng/µL and used for subsequent RT-qPCR. RT-qPCR was performed using a LightCycler 480 SYBR Green I Master real-time fluorescent PCR instrument with three replicates per well. Glyceraldehyde-3-phosphate dehydrogenase (GAPDH) served as the reference gene. The primer sequences are shown in Additional file 5: Table S3. The 2^−ΔΔCt^ method was used to quantify the relative expression levels of target genes [31, 42, 43].

### Western blot analysis

Total protein was extracted from tissues or cells using high-efficiency Radio Immunoprecipitation Assay (RIPA) lysis solution (C0481, Sigma-Aldrich) containing 1% protease inhibitor and 1% phosphatase inhibitor (Beyotime). The protein concentration of each sample was determined using the bicinchoninic acid (BCA) protein quantification kit (23227, Thermo Fisher Scientific, Rockford, IL). Proteins were separated by sodium dodecyl sulfate–polyacrylamide gel electrophoresis (SDS-PAGE) and then electrotransferred to the polyvinylidene fluoride (PVDF; Millipore, Billerica, MA) membrane. After blocking with 5% bovine serum albumin (BSA) for 1 h at room temperature, the membrane was probed with the diluted antibodies, rabbit anti-ERK1/2 antibody (1:10,000, ab184699, Abcam), rabbit anti-AKT2 antibody (1:500, ab131168, Abcam) and rabbit anti-GAPDH antibody (1:500, ab9485, Abcam) at 4 °C overnight. The next day, the membrane was washed with 1 × TBST and then incubated with horseradish peroxidase (HRP)-labeled goat anti-rabbit IgG secondary antibody (1:5000, ab205718, Abcam) for 1.5 h at room temperature. The ECL reagent-visualized blots were quantified in greyscale using ImageJ 1.48 u software (Bio-Rad, Hercules, CA), with GAPDH as the internal reference [43]. Each experiment was repeated three times to calculate the mean value ± standard deviation.

### In vivo experiment protocols

Sixty-four male Wistar rats (6 weeks old) were purchased from Vital River Laboratory Animal Technology Co., Ltd. (Beijing, China) and housed in a specific pathogen-free (SPF) environment with 12-h light–dark cycles and humidity of 60–65% at 22–25 °C, with free access to food and water. The experiment was conducted after acclimatization for one week. The femoral defects were established. A 3 cm skin incision was made on the center of the left lateral femur, and the periosteum on the metaphyseal surface of the distal femur was dissected. Using a trephine with normal saline irrigation, a transcortical bone defect of 3 mm diameter was created in the anteroposterior direction of the left femoral metaphysis. The area was rinsed with normal saline to manage bone debris and heat from drilling. In vivo micro-CT scanning was performed on the 56^th^ day after surgery, and the repair of the femoral defects was evaluated by combining it with histological analysis [44, 45]. As illustrated in Additional file 2: Fig. S1, the results indicate that the rat model was successfully established.

The rats were divided into 8 groups (*n* = 8): (1) Blank group (rats without treatment); (2) PMMA group (PMMA was implanted into the rat femoral defect); (3) ES group (ES was implanted into the rat femoral defect); (4) PMMA@ES (PMMA@ES was implanted into the rat femoral defect); (5) PMMA@ES + sh-NC (PMMA@ES and sh-NC plasmids were implanted into the rat femoral defect); (6) PMMA@ES + sh-ATF2 (PMMA@ES and sh-ATF2 plasmids were implanted into the rat femoral defect); (7) PMMA@ES + oe-NC (PMMA@ES and oe-NC plasmids were implanted into the rat femoral defect); (8) PMMA@ES + oe-ERK1/2 (PMMA@ES and oe-ERK1/2 plasmids were implanted into the rat femoral defect). The entire femoral bone was taken and underwent micro-computed tomography (CT) scanning. All specimens were washed 3 times with PBS and fixed in 4% paraformaldehyde at 4 °C for 48 h. Animal experiments were approved by the medical ethics committee of the Third Hospital of Hebei Medical University (No. z2021-007-2).

### Immunohistochemistry (IHC)

Paraffin-embedded sections of rat femoral bone tissues were collected. The samples were subjected to antigen retrieval after being immersed in 3% methanol H_2_O_2_ for 20 min. Next, the tissue sections were added with normal goat serum blocking solution (C-0005, Shanghai Haoran Biotechnology Co. Shanghai, China) at room temperature for 20 min. The tissue sections were added with primary antibodies, rabbit anti-mouse RUNX2 (SAB1403638, 1:500, Sigma), OCN (AB10911, 1:500, Sigma), ATF2 (ab32160, 1:10,000, Abcam) and ERK1/2 (ab17942, 1:100, Abcam) at 4 °C overnight. Subsequently, the sections were incubated with the secondary antibody goat anti-rabbit IgG (ab6785, 1:1000, Abcam) at 37 °C for 20 min, followed by incubation with HRP-labeled working solution of streptomyces ovalbumin (Imunbio Co., Ltd., Beijing, China) at 37 °C for 20 min. The sections were developed by DAB (ST033, Guangzhou Weijia Technology Co., Ltd., Guangzhou, China), and counter-stained with hematoxylin (PT001, Shanghai Bogoo Biotechnology Co., Ltd., Shanghai, China), and blued in 1% ammonia. Next, the sections were observed and photographed under a microscope. Five high magnification fields were randomly selected for each section, with 100 cells in each field. The sections were examined by an experienced pathologist [46, 47].

### Micro-CT scanning

A micro-CT scanner (SCANCO µCT50, Muttenz, Switzerland) was used to detect the newly formed bones in the femoral defects of the rat animal model. A Mimics software (Mimics 17.0, Materialise, Leuven, Belgium) was employed to obtain three-dimensional images and calculate the bone mineral density (BMD) and bone volume/tissue volume (BV/TV) of the new bone-like tissues (BV/TV = bone surface area/tissue volume; the higher the BMD, the higher the CT value). Micro-CT can observe overall bone volume changes and quantitative indicators of trabecular bone microstructure within the bone, which combine trabecular microstructural indicators, cortical bone indicators, and bone volume to evaluate the role of bone strength and bone mass during osteoporosis.

### Statistical analysis

Data were analyzed using the SPSS 22.0 software (IBM, Armonk, NY). All measurement data are presented as mean ± standard deviation. The normality and homogeneity of variance were conducted. The data conforming to a normal distribution and homogeneous variance between two groups were analyzed by unpaired *t*-test, and the comparison of data among multiple groups was tested by one-way analysis of variance (ANOVA), followed by Tukey’s post hoc test*. p* < 0.05 was considered statistically significant.

## Results

### ATF2 may be a key gene involved in the differentiation of BMSCs into osteoblasts

**Fig. 1:**
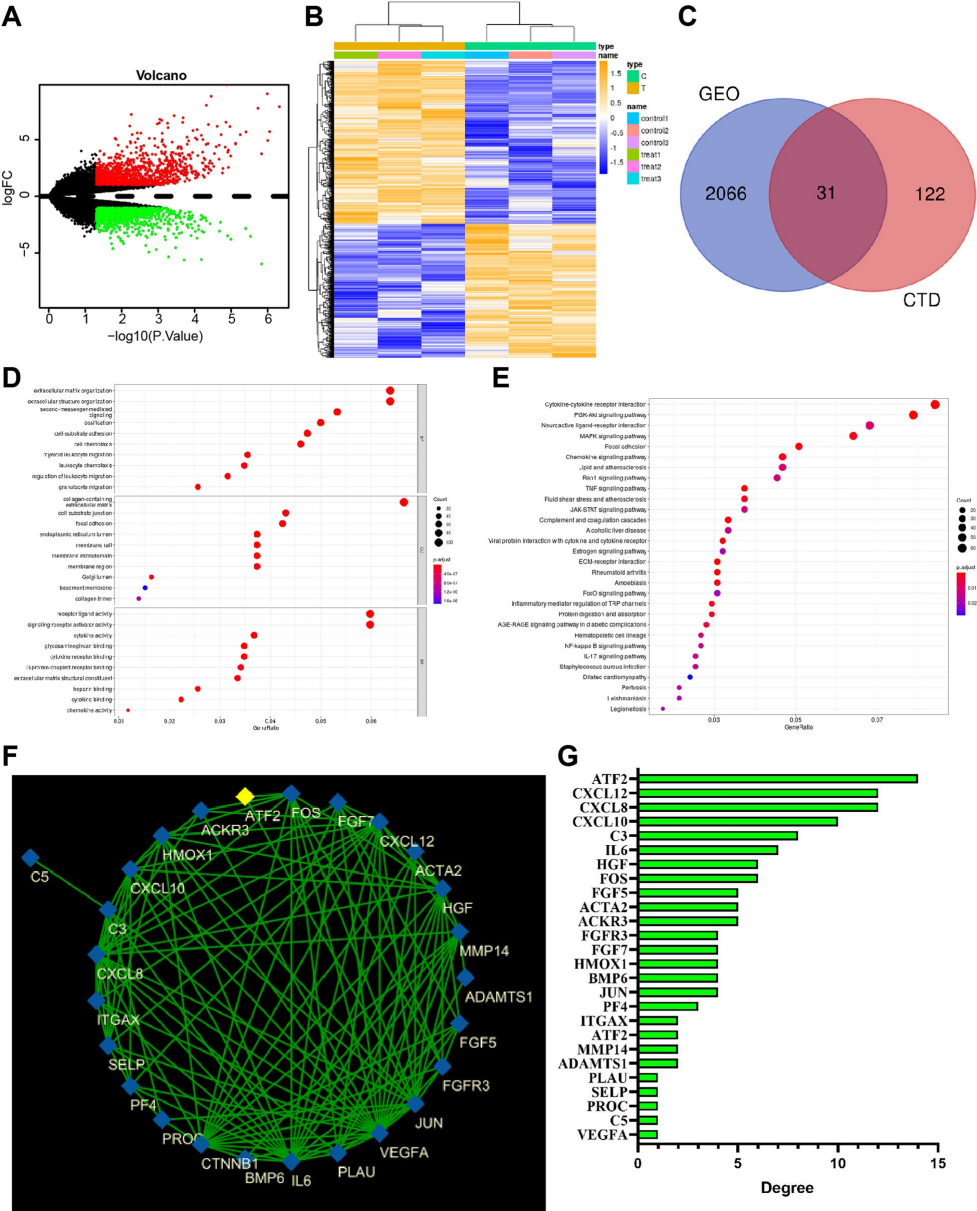
Identification of key genes involved in the osteogenic differentiation of BMSCs. **A** Volcano map of differential analysis for microarray data GSE9451. Red dots indicate upregulated genes, green dots indicate downregulated genes and black dots indicate genes without significant difference (BMSCs group, *n* = 3; BMSCs_osteogenesis group, *n* = 3). **B** The heat map of differential analysis for microarray data GSE9451 dataset (BMSCs group, *n* = 3; BMSCs_osteogenesis group, *n* = 3). **C** Venn map of the intersection between CTD database and microarray data GSE9451. **D** GO enrichment analysis for candidate target genes. Abscissa indicates GeneRatio. **E** KEGG enrichment analysis for candidate target genes. Abscissa indicates GeneRatio. **F** Protein interaction among the 31 candidate genes. Nodes represent genes, and edges represent the interactions between genes. **G** Candidate target genes according to Degree values

The BMSC osteogenic differentiation-related microarray data GSE9451 was downloaded from the GEO database, and 2096 DEGs in osteogenic differentiation of BMSCs (1151 upregulated and 945 downregulated) were screened with |log2FC|> 2 and *p* < 0.05 as the threshold (Fig. 1A, B). The CTD database retrieved 153 target genes, and 31 intersected target genes were obtained using the Venn diagram (Fig. 1C). Further, GO enrichment analysis of the candidate target genes revealed that they were mainly involved in biological processes, such as PI3K-Akt signaling pathway, Neuroactive ligand-receptor interaction, and cell-substrate adhesion (Fig. 1D, E). The 31 candidate target genes associated with ES-induced differentiation of BMSCs into osteoblasts were imported into the STRING database, with the species restricted to humans, to obtain protein interaction relationships and further imported into Cytoscape software to construct a protein interaction network (Fig. 1F). Ranking according to the number of connections between genes (Degree value), it is found that ATF2 ranked the first (Fig. 1G).

Numerous studies have demonstrated that upregulated ATF2 promotes osteogenic differentiation of MSCs [48, 49]. Thus, it can be speculated that ATF2 may be a key gene involved in differentiating BMSCs into osteoblasts.

### ATF2 activates transcription of miR-335-5p and represses ERK1/2 during the osteogenic differentiation of BMSCs

**Fig. 2:**
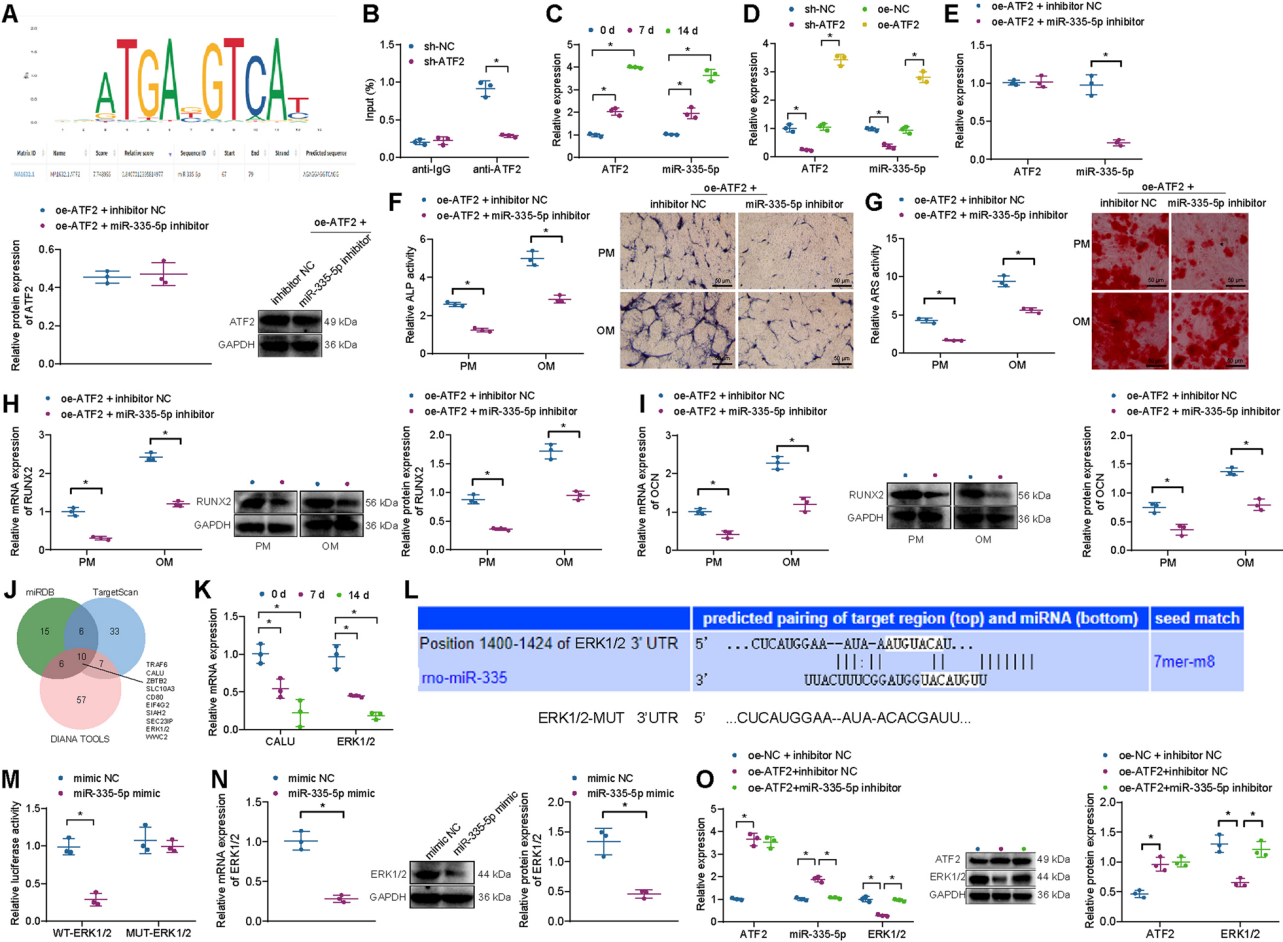
Effects of ATF2 on the osteogenic differentiation of BMSCs. **A** JASPAR website was used to predict the binding site of the ATF2 transcription factor to miR-335-5p. **B** ChIP assay was used to detect ATF2 enrichment in the promoter region of miR-335-5p. **C** RT-qPCR for ATF2 and miR-335-5p expression in BMSCs after 0, 7, and 14 days of osteogenic differentiation. **D** RT-qPCR for miR-335-5p expression in BMSCs after exogenous interference with ATF2. **E** Western blot analysis and RT-qPCR were used to detect miR-335-5p and ATF2 expression in BMSCs treated with oe-ATF2 or in combination with miR-335-5p inhibitor. **F** ALP staining and quantification of BMSCs treated with oe-ATF2 or in combination with miR-335-5p inhibitor in PM and OM. **G** ARS staining and quantification of BMSCs treated with oe-ATF2 or in combination with miR-335-5p inhibitor in PM and OM.**H** Western blot analysis and RT-qPCR were used to detect RUNX2 expression in BMSCs treated with oe-ATF2 or combined with miR-335-5p inhibitor in PM and OM. **I** Western blot analysis, and RT-qPCR were used to detect OCN expression in BMSCs treated with oe-ATF2 or in combination with miR-335-5p inhibitor in PM and OM. **J** Venn diagram of the miRDB, TargetScan, and DIANA TOOLS databases results. **K** RT-qPCR for CALU and ERK1/2 expression in BMSCs after 0, 7, and 14 days of osteogenic differentiation. **L** TargetScan database was used to predict the target binding sites of miR-335-5p to miR-335-5p to ERK1/2. **M** Dual-luciferase reporter assay was used to verify the binding of miR-335-5p to ERK1/2. **N** Western blot analysis, and RT-qPCR were used to detect ERK1/2 expression in BMSCs transfected with miR-335-5p mimic. **O** Western blot analysis and RT-qPCR were used to detect ATF2, miR-335-5p, and ERK1/2 expression in BMSCs treated with oe-ATF2 alone or combined with miR-335-5p inhibitor. *p < 0.05. Cell experiments were repeated three times

Existing evidence has shown that miR-335-5p can promote bone regeneration [25]. Meanwhile, based on the above results, we speculate that ATF2 may play a corresponding regulatory function through miR-335-5p. We employed the JASPAR website and predicted that ATF2 might act as a transcription factor to activate the transcription of miR-335-5p and promote its expression (Fig. 2A). To verify whether ATF2 promotes the osteogenic differentiation of BMSCs through miR-335-5p, we first designed two ATF2 knockdown sequences. We found that sh-ATF2-2 had an optimal knockdown efficiency according to RT-qPCR and Western blot analysis results, which was used for subsequent ChIP assay (Additional file 3: Fig. S2A, B).

Subsequently, ChIP assay results showed that the knockdown of ATF2 resulted in a significant decrease in the enrichment levels of ATF2 at the miR-335-5p gene promoter region (Fig. 2B). The RT-qPCR detection results showed that the expression levels of both ATF2 and miR-335-5p gradually increased during the osteogenic differentiation of BMSCs at 0, 7, and 14 days (Fig. 2C). Furthermore, compared to the sh-NC group, the expression levels of ATF2 and miR-335-5p were significantly downregulated in the sh-ATF2 group, and compared to the oe-NC group, the expression levels of ATF2 and miR-335-5p were significantly upregulated in the oe-ATF2 group in BMSCs (Fig. 2D). These results indicate that ATF2 may act as a transcription factor to activate the transcription of the miR-335-5p gene and promote its expression during the osteogenic differentiation of BMSCs.

Additionally, the results of RT-qPCR and Western blot analysis suggested no significant changes in the expression of ATF2, while the expression of miR-335-5p was reduced in BMSCs upon treatment with oe-ATF2 + miR-335-5p inhibitor compared to treatment with oe-ATF2 + inhibitor NC (Fig. 2E). In the PM and OM, ALP activity and extracellular matrix mineralization were attenuated, and the expression of RUNX2 and OCN was downregulated in response to co-treatment with oe-ATF2 and miR-335-5p inhibitor relative to individual treatment with oe-ATF2 (Fig. 2F–I). These data indicate that the knockdown of miR-335-5p can restore the osteogenic differentiation of BMSCs induced by overexpression of ATF2.

We further explored the downstream pathway of miR-335-5p and predicted the downstream target genes of miR-335-5p by miRDB, TargetScan, and DIANA TOOLS databases. The miRDB database screening criterion was a Target Score of ≥ 90. The TargetScan database screening criterion had a Total context++ score of ≤  − 0.30, and the DIANA TOOLS database screening criterion had a miTG score ≥ 0.95. The results of the three databases were intersected to obtain 10 genes (ERK1/2, CALU, ZBTB2, SLC10A3, CD80, EIF4G2, SIAH2, SEC23IP, BCORL1, and WWC2) (Fig. 2J). Among them, only CALU and ERK1/2 expressions were differentially expressed during the osteogenic differentiation of BMSCs, and both of them gradually decreased (Fig. 2K and Additional file 4: Fig. S3).

It has been reported that miRNAs of BSMC-PMMA@ES-derived miRNA delay the development of disc degeneration by targeting ERK1/2 [50]. miR-335-5p can target ERK1/2 [29], but its role in the osteogenic differentiation of BMSCs remains to be further explored. Based on the specific binding sites predicted from the TargetScan database (Fig. 2L). The binding of miR-335-5p to ERK1/2 was further validated by dual-luciferase reporter assay, respectively. It was found that miR-335-5p had a better target relationship with ERK1/2 respectively (Fig. 2M). Meanwhile, RT-qPCR and Western blot analysis demonstrated that ERK1/2 mRNA and protein expression was diminished upon miR-335-5p mimic transfection (Fig. 2N). Thus, miR-335-5p may target ERK1/2 and inhibit ERK1/2 expression during the osteogenic differentiation of BMSCs.

To elucidate the regulatory relationship between ATF2-miR-335-5p/ERK1/2 signaling axis during osteogenic differentiation of BMSCs, ATF2, and miR-335-5p expression were interfered with in BMSCs. The results of RT-qPCR and Western blot analysis displayed that transfection with oe-ATF2 upregulated the expression of ATF2 and reduced the expression of ERK1/2 without affecting the expression of miR-335-5p. However, oe-ATF2 combined with miR-335-5p inhibitor exerted no effects on the ATF2 expression, downregulated miR-335-5p expression, and upregulated ERK1/2 expression (Fig. 2O), suggesting that knockdown of miR-335-5p expression could restore the inhibition of ERK1/2 expression by overexpression of ATF2.

Overall, these results indicate that the expression of ATF2 and miR-335-5p was elevated, and the expression of ERK1/2 was downregulated during the osteogenic differentiation of BMSCs. ATF2 may target the expression of ERK1/2 by activating the transcription of miR-335-5p during the osteogenic differentiation of BMSCs.

### ATF2 induces the osteogenic differentiation of BMSCs by downregulating the expression of ERK1/2

**Fig. 3:**
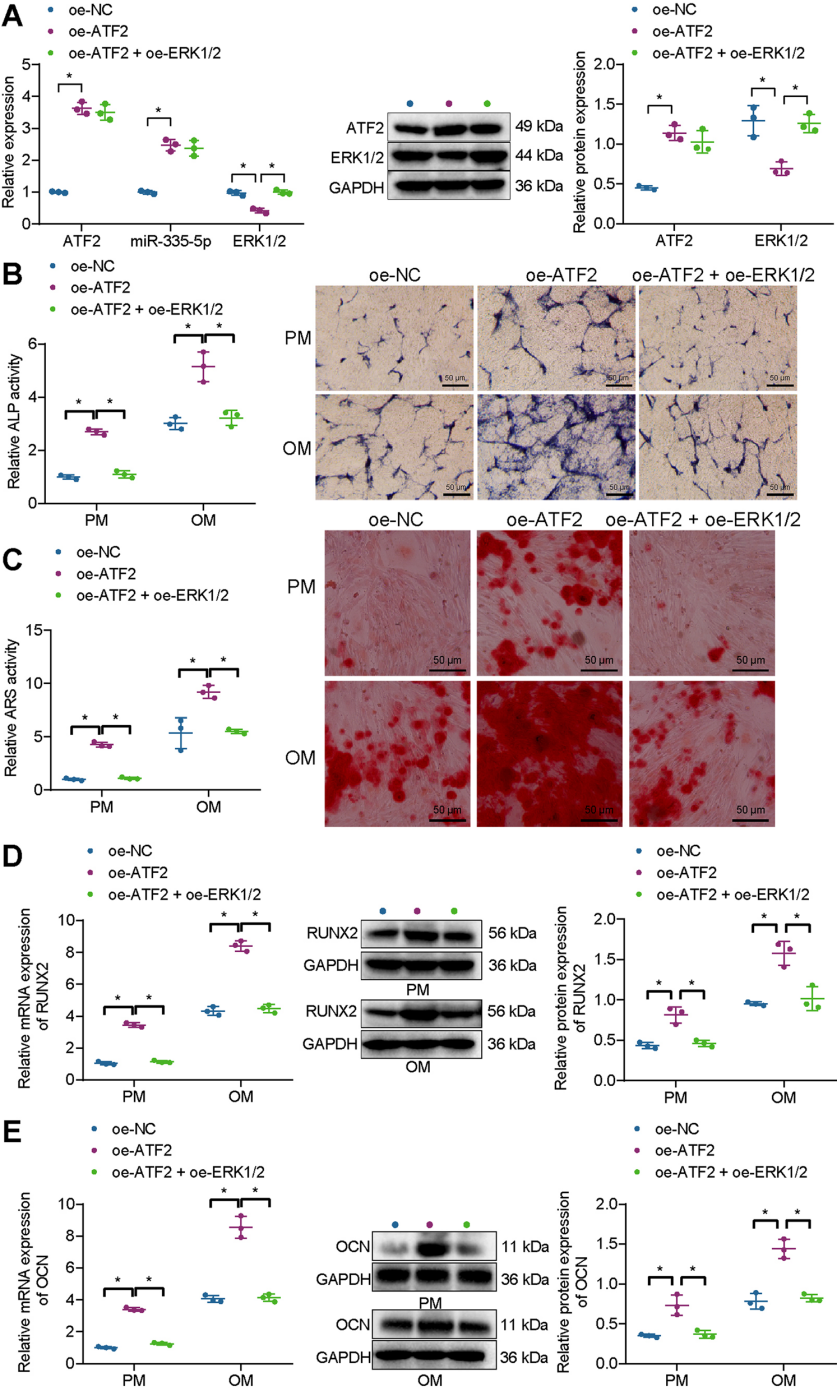
Effects of ATF2 on the osteogenic differentiation of BMSCs via regulation of ERK1/2. BMSCs were treated with oe-ATF2 or in combination with oe-ERK1/2. **A** Western blot analysis and RT-qPCR were used to detect miR-335-5p, ERK1/2, and ATF2 expression in BMSCs. **B** ALP staining for BMSCs in PM and OM. **C** ARS staining for BMSCs in PM and OM. **D** Western blot analysis and RT-qPCR were used to detect RUNX2 expression in BMSCs in PM and OM. **E** Western blot analysis and RT-qPCR were used to detect OCN expression in BMSCs in PM and OM. *p < 0.05. Cell experiments were repeated three times

We then investigated whether ATF2 promotes the osteogenic differentiation of BMSCs by activating the transcription of miR-335-5p, thereby reducing the expression of ERK1/2. BMSCs were treated with oe-ATF2 or combined with oe-ERK1/2. The results of RT-qPCR and Western blot analysis displayed an increase of ATF2 and miR-335-5p expression and a decline of ERK1/2 expression in response to oe-ATF2; conversely, further, oe-ERK1/2 treatment failed to alter ATF2 and miR-335-5p expression but upregulated ERK1/2 expression. In addition, ALP staining, alizarin red staining, RT-qPCR, and Western blot analysis results showed that in PM and OM, overexpression of ATF2 strengthened ALP activity and extracellular matrix mineralization and increased expression of RUNX2 and OCN, indicating that overexpression of ATF2 could induce osteogenic differentiation of BMSCs. Moreover, oe-ATF2 combined with oe-ERK1/2 weakened ALP activity and extracellular matrix mineralization and reduced the expression of RUNX2 and OCN (Fig. 3A–E).

The obtained data revealed that overexpression of ERK1/2 can antagonize the osteogenic differentiation of BMSCs induced by ATF2.

### PMMA@ES significantly promotes the osteogenic differentiation of BMSCs

**Fig. 4:**
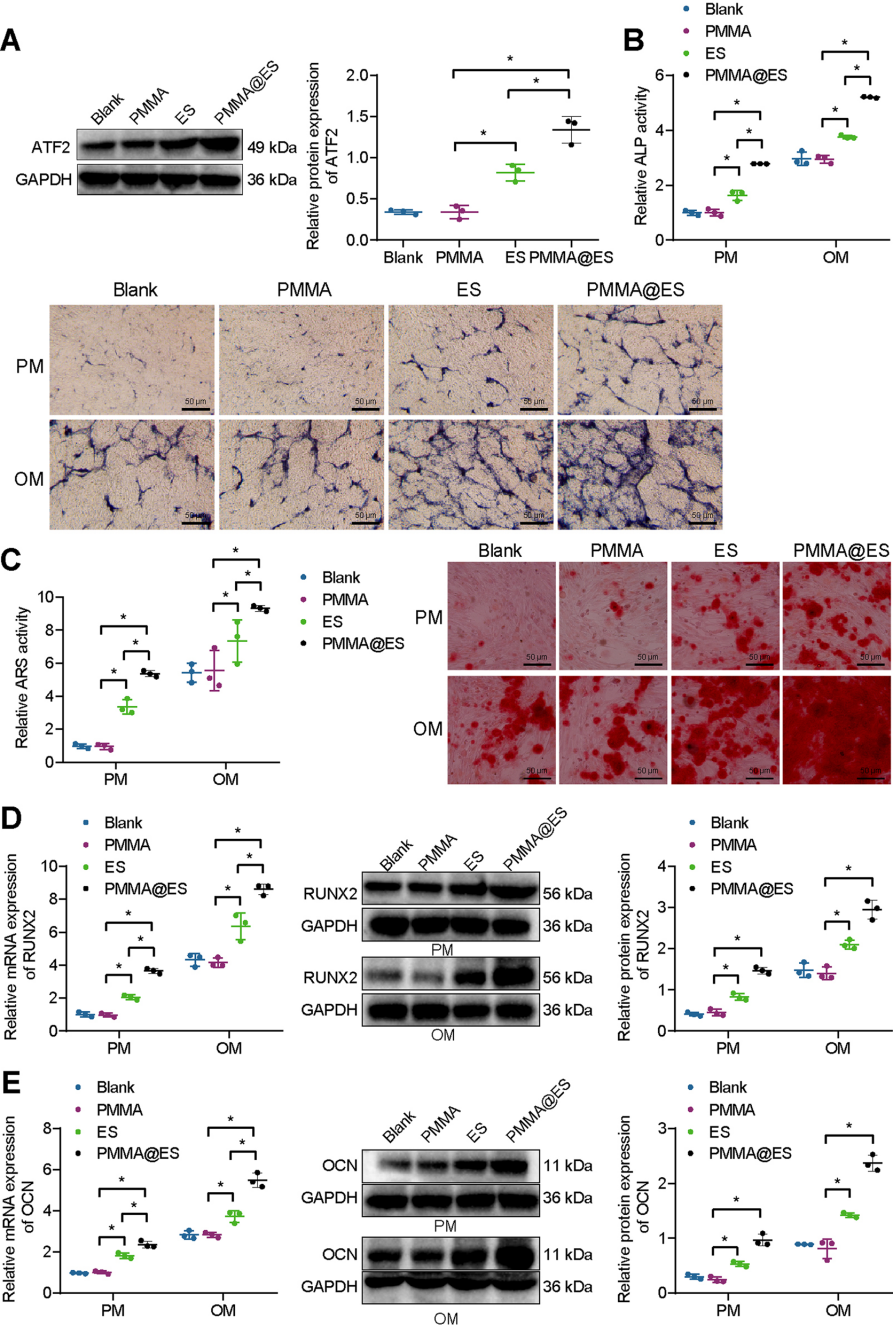
Effects of PMMA@ES on the osteogenic differentiation of BMSCs. **A** Western blot analysis for ATF2 protein levels in BMSCs treated with PMMA, ES, or PMMA@ES. **B** ALP staining and quantification of BMSCs treated with PMMA, ES, or PMMA@ES in PM and OM. **C** ARS staining and quantification of BMSCs treated with PMMA, ES, or PMMA@ES in PM and OM. **D** Western blot analysis and RT-qPCR were used to detect RUNX2 expression in BMSCs treated with PMMA, ES, or PMMA@ES in PM and OM. **E** Western blot analysis and RT-qPCR were used to detect OCN expression in BMSCs treated with PMMA, ES, or PMMA@ES in PM and OM. *p < 0.05. Cell experiments were repeated three times

Previous data have shown that PMMA@ES can promote the repair of tibial defects [51]. Therefore, we speculate that PMMA@ES may play a role in regulating the osteogenic differentiation of BMSCs. To investigate the effect of PMMA@ES on the osteogenic differentiation of BMSCs, we treated BMSCs with PMMA, ES, and PMMA@ES. The results of Western blot analysis showed no changes in the expression of ATF2 in BMSCs treated with PMMA and without treatment. Compared with BMSCs treated with PMMA, the expression of ATF2 was increased in BMSCs treated with ES or PMMA@ES, with the latter exhibiting a higher expression of ATF2 (Fig. 4A). These results suggested that PMMA@ES could effectively increase ATF2 expression in BMSCs.

BMSCs were cultured using PM, and OM, respectively, and ALP activity was measured using a p-NPP solution after 7 days. Chemical nodule formation was observed after 14 days by alizarin red staining, and RT-qPCR and Western blot analysis measured the expression of the genes RUNX2 and OCN. No changes were observed in ALP staining and activity, extracellular matrix mineralization, and RUNX2 and OCN expression in BMSCs treated with PMMA and without treatment. In both PM and OM, compared with BMSCs treated with PMMA, ALP staining, and activity, extracellular matrix mineralization was enhanced, and RUNX2 and OCN expression was upregulated in BMSCs treated with ES or PMMA@ES. These phenomena were more obvious in BMSCs treated with PMMA@ES than BMSCs treated with ES (Fig. 4B–E).

### PMMA@ES induces femoral defect regeneration in rats with femoral bone defects

Rat models of femoral bone defects were successfully constructed. Rat models without any treatment (Blank group) served as a control, and the remaining rat models were treated with PMMA, ES, PMMA@ES, PMMA@ES + sh-NC, PMMA@ES + oe-NC, PMMA@ES + sh-ATF2 and PMMA@ES + oe-ERK1/2.

**Fig. 5:**
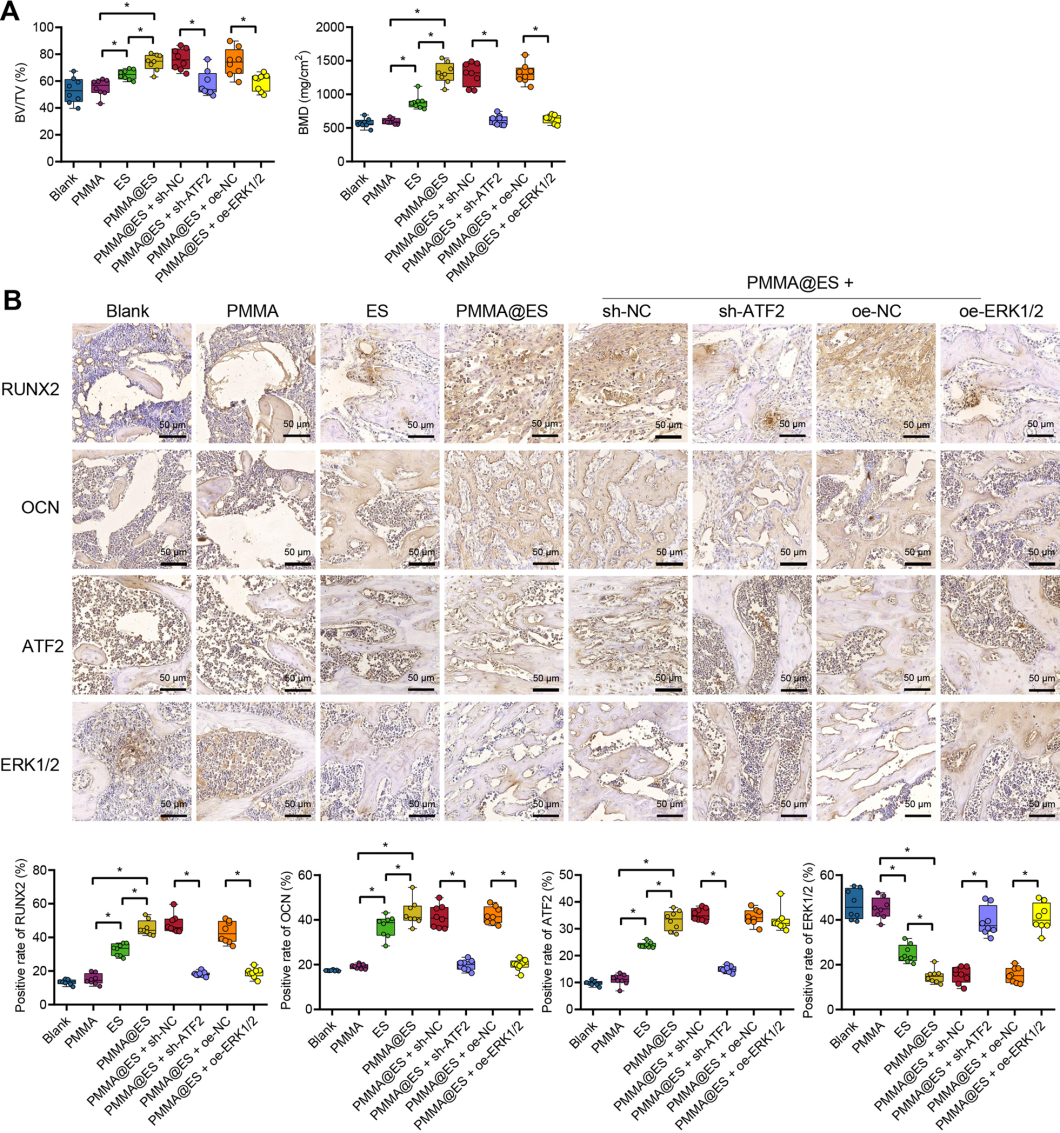
Effects of PMMA@ES on femoral defect regeneration in rats via the ATF2/miR-335-5p/ERK1/2 axis. Rats with femoral defects were treated with PMMA, ES, or PMMA@ES (*n* = 8). **A** micro-CT scanning quantitative results of the site of the femoral defect in rats. **B** Immunohistochemical staining for positive expression of RUNX2, OCN, ATF2, and ERK1/2 at the new bone formation site of rats with femoral defects. Dark-brown granules indicating positive staining are marked by black arrows. *p < 0.05

Micro-CT scanning images showed no significant differences in new bone formation, BV/TV ratio, and BMD between the Blank and PMMA groups. More new bone formation and significantly greater BV/TV ratio and BMD were found in rats with femoral bone defects treated with ES than in rats without treatment. However, the effects of ES were more evident in rats with femoral bone defects treated with PMMA@ES. In addition, compared to PMMA@ES treatment, new bone formation, BV/TV ratio, and BMD presented no changes in rats treated with PMMA@ES + sh-NC and PMMA@ES + oe-NC. In response to PMMA@ES + sh-ATF2 treatment, a reduction was found in new bone formation, BV/TV ratio, and BMD in rats versus PMMA@ES + sh-NC treatment. Similar results in these parameters were obtained in response to PMMA@ES + oe-ERK1/2 treatment versus PMMA@ES + oe-NC treatment (Fig. 5A). In addition, immunohistochemical staining exhibited no significant difference in the positive expression of ATF2, RUNX2, OCN, and ERK1/2 in rats with femoral bone defects treated with PMMA and without any treatment. The rats with femoral bone defects treated with ES showed a higher range and intensity of the ATF2, RUNX2, and OCN stained granules around the nucleus and in the cytoplasm of osteoblasts yet a lower intensity of ERK1/2 than rats treated with PMMA. In contrast, the positive expression of ATF2, RUNX2, and OCN in the rats with femoral bone defects treated with PMMA@ES was enhanced, while the positive expression of ERK1/2 was reduced relative to ES treatment alone. PMMA@ES + sh-NC or PMMA@ES + oe-NC did not alter the positive expression of ATF2, ERK1/2, RUNX2, and OCN compared to PMMA@ES. Relative to PMMA@ES + sh-NC, PMMA@ES + sh-ATF2 reduced the positive expression of ATF2, RUNX2, and OCN while augmenting that of ERK1/2. Furthermore, PMMA@ES + oe-ERK1/2 caused no effects on the positive expression of ATF2, increased ERK1/2 positive expression, and decreased RUNX2 and OCN positive expression (Fig. 5B).

Therefore, it can be concluded that PMMA@ES could induce femoral defect regeneration and promote femoral defect repair in rats, which may be associated with the ATF2/miR-335-5p/ERK1/2 axis.

## Discussion

PMMA-based acrylic bone cement is commonly used to fix bone and metallic implants in orthopedic procedures [52, 53], but the underlying mechanistic basis of osteogenesis remains largely unknown. This study reported a possible mechanism that PMMA@ES-induced ATF2 overexpression activates miR-335-5p to target ERK1/2, thereby promoting the osteogenic differentiation and femoral defect regeneration in BMSCs and ultimately enhancing the repair of femoral defects in rats.

A recent study has confirmed that ATF2 promotes the homing and differentiation of BMSC to neuronal cells [54]. Moreover, phosphorylation (activated form) of ATF2 enhances the differentiation of BMSCs to brown adipocytes [55]. In addition, ATF2 also induces the differentiation of stem cells towards adipocytes [56]. These findings are consistent with our study, which confirmed that ATF2 may be a key gene in differentiating BMSCs into osteoblasts.

In vitro cell assays in the current study demonstrated that PMMA@ES effectively elevated ATF2 expression in BMSCs, thereby promoting the osteogenic differentiation of BMSCs. Numerous studies have indicated that PMMA greatly affects BMSC proliferation and osteogenic differentiation for internal fixation of fractures in osteoporotic patients [34, 57]. ES can promote osteogenic repair [58], and ATF2 has also been reported to be associated with a pro-differentiation effect [55]. These studies share the same findings with our study.

ATF2 has been reported to activate the transcription of various RNAs, including lncRNAs and miRNAs [59, 60]. Previous studies have exhibited that miR-335-5p can participate in the occurrence and development of tumors by regulating the ERK pathway [61, 62]. However, there are few studies about miR-335-5p activation by AFT2 targeted by miR-335-5p. Moreover, in vitro experiments in the present study revealed that ATF2 activates the transcription of miR-335-5p during the osteogenic differentiation of BMSCs, thereby targeting ERK1/2. Our findings further elucidated the important role of the ATF2/miR-335-5p/ERK1/2 axis in the osteogenic differentiation of BMSCs, which was also verified by the previous studies [54, 55].

In addition, the obtained data confirmed that PMMA@ES upregulated ATF2 expression and activated the miR-335-5p/ERK1/2 axis to induce osteogenic differentiation of BMSCs and femoral defect regeneration in rats. ATF2 has been confirmed to promote differentiation and activate the transcriptional processes of multiple RNAs, including miRNAs [59, 60]. Activation of miR-335-5p can enhance the osteogenic differentiation of BMSCs [25], consistent with our findings. ERK pathway has been validated to have a strong osteogenic role. Further, FGF9 can inhibit osteogenesis in BMSCs by promoting the ERK pathway [63].

## Conclusion

**Fig. 6:**
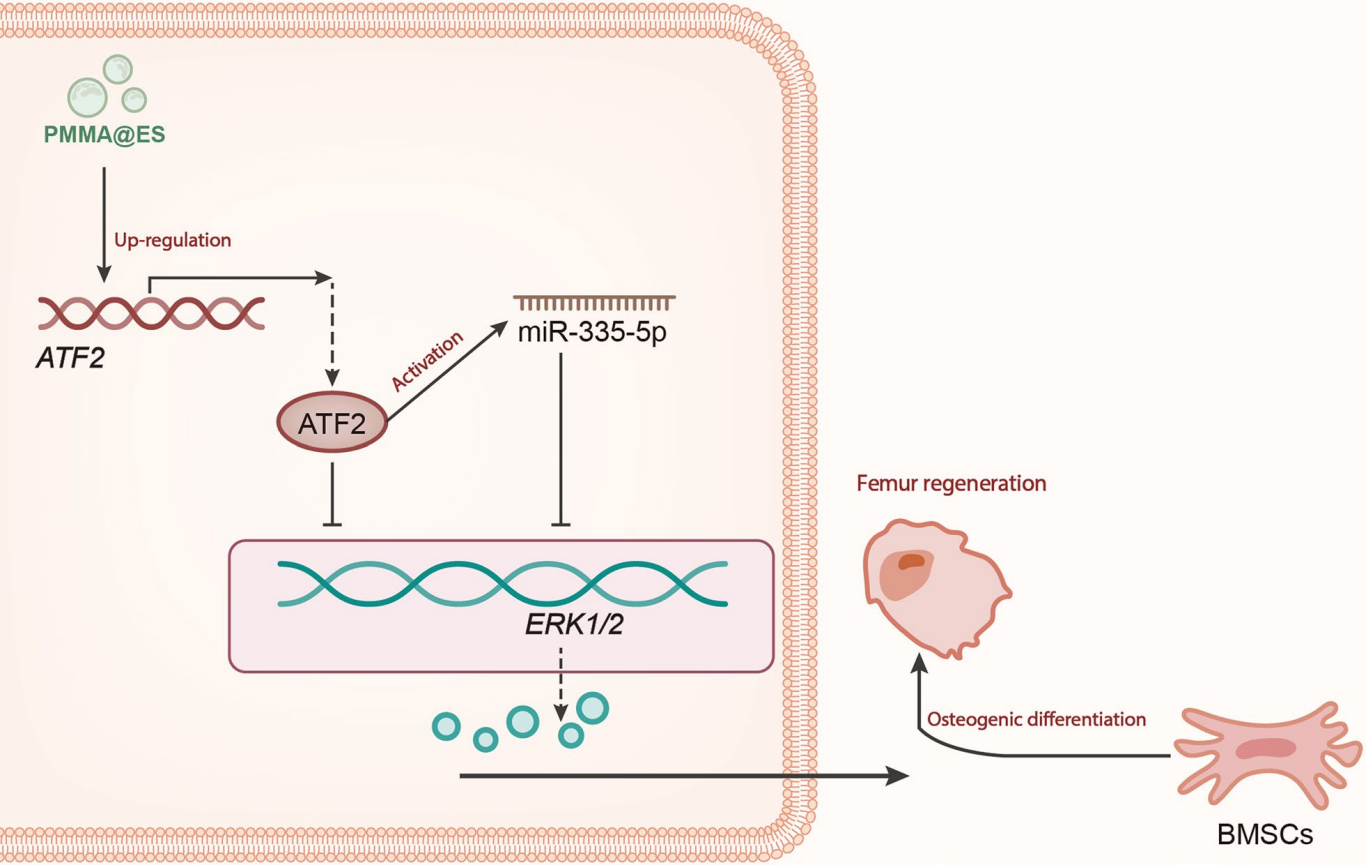
Schematic representation of the molecular mechanism of PMMA@ES in femoral defects. PMMA@ES-induced osteogenic differentiation of BMSCs involved in femoral defect regeneration by elevating ATF2 expression and activating the miR-335-5p/ERK1/2 axis

In conclusion, the findings of our study demonstrated that PMMA@ES could upregulate ATF2 and activate the transcription of miR-335-5p to promote osteogenic differentiation and femoral defect regeneration in BMSCs by targeting ERK1/2, ultimately promoting femoral bone defect repair in rats (Fig. 6). The above findings are beneficial to further exploration of the specific molecular mechanisms of PMMA@ES in the osteogenic differentiation of BMSCs and femoral defect regeneration, providing new evidence for the application of PMMA@ES in bone defects. However, our study only confirmed the pro-regenerative effect of PMMA@ES in vivo, while the effects of the miR-335-5p/ERK1/2 axis on femoral defect regeneration in vivo experiments have not been validated. Moreover, it is unknown which factors regulate the low expression of ATF2 in osteogenic differentiation and bone injury of BMSCs, which should be further explored in future studies.

### Supplementary Information

**Additional file 1**. Supplementary figures.

**Additional file 2: Figure S1** Representative micro-CT images of the femurs in rats following femoral defect models.

**Additional file 3: Figure S2** Detection of knockdown efficiency of two sh-ATF2 sequences by Western blot analysis and RT-qPCR. *p < 0.05. Cell experiments were repeated three times.

**Additional file 4: Figure S3** RT-qPCR detection of changes in the expression of ten target genes (ZBTB2, SLC10A3, CD80, EIF4G2, SIAH2, SEC23IP, BCORL1, and WWC2) after 0, 7, 14 days of BMSC osteogenic differentiation. Cell experiments were repeated three times.

**Additional file 5: Table S1** shRNA sequences. **Table S2** The qPCR primer sequences in ChIP. **Table S3** RT-qPCR primer sequences

## Data Availability

The datasets generated and/or analyzed during the current study are available from the corresponding author upon reasonable request.
