# Supplementary material for: ATF2-driven osteogenic activity of enoxaparin sodium-loaded polymethylmethacrylate bone cement in femoral defect regeneration
Source: J Orthop Surg Res. 2023 Aug 31;18:646. doi: 10.1186/s13018-023-04017-8 (PMC10470168; doi:10.1186/s13018-023-04017-8)
Supplement: Supplementary file 1 — Additional file 1. Supplementary figures. [file 13018_2023_4017_MOESM1_ESM.docx]

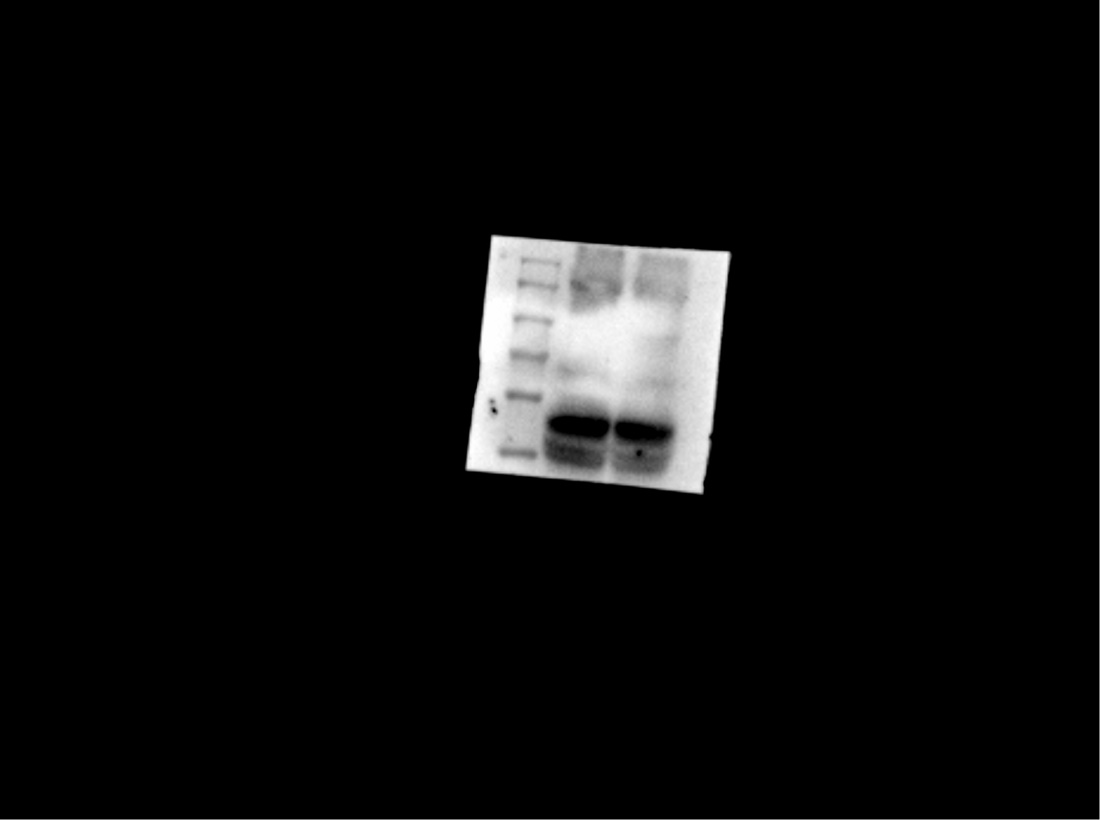


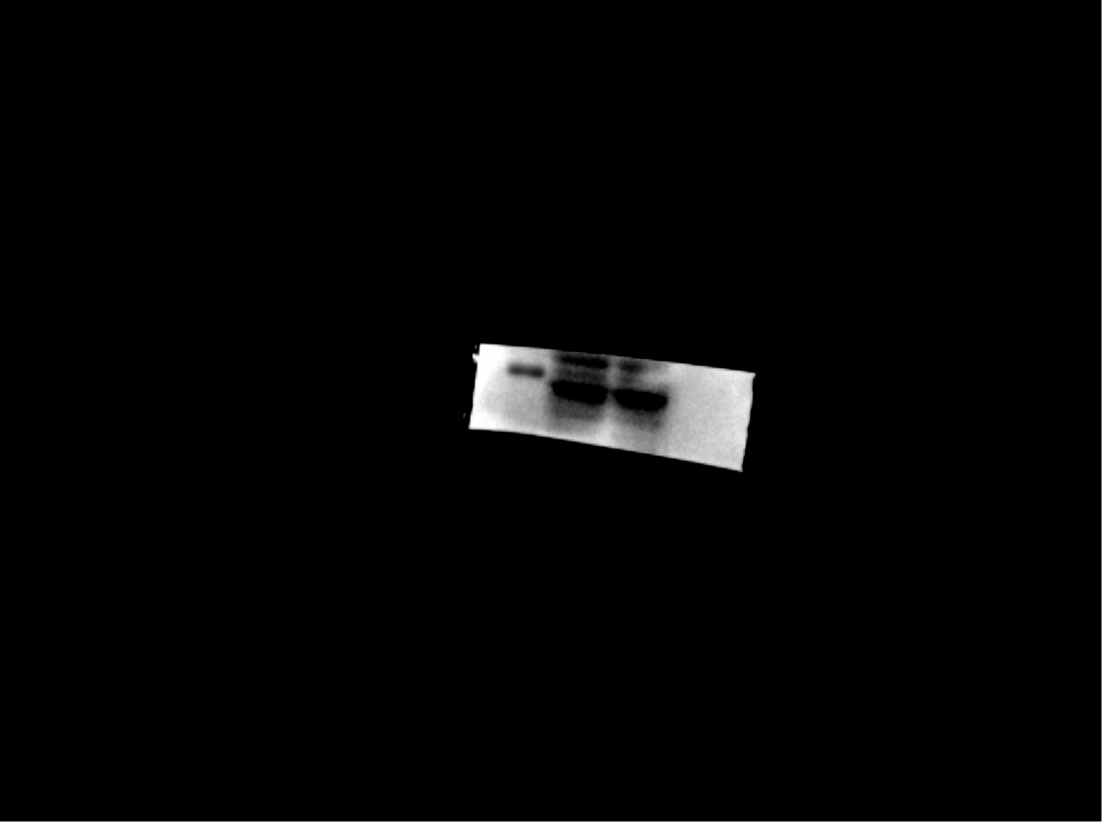


Figure 2E


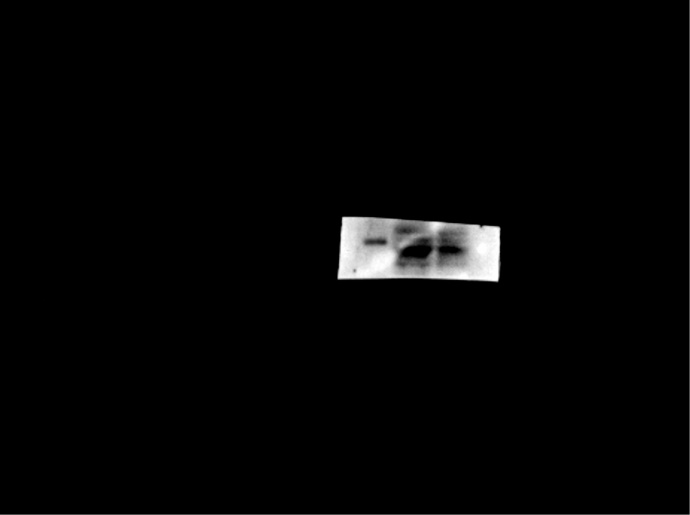


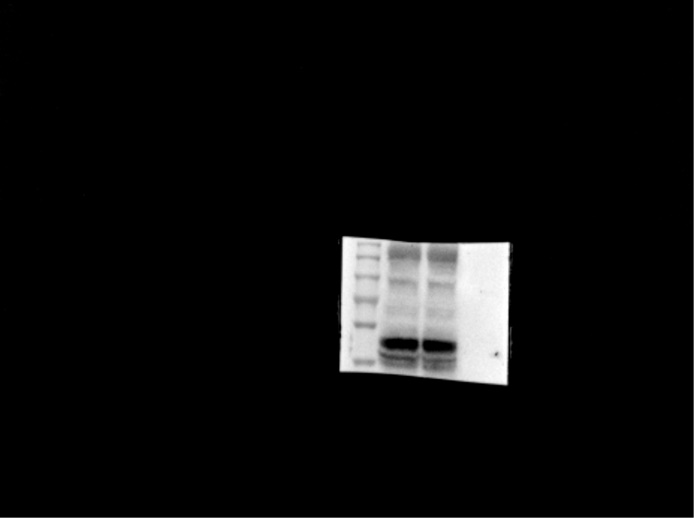


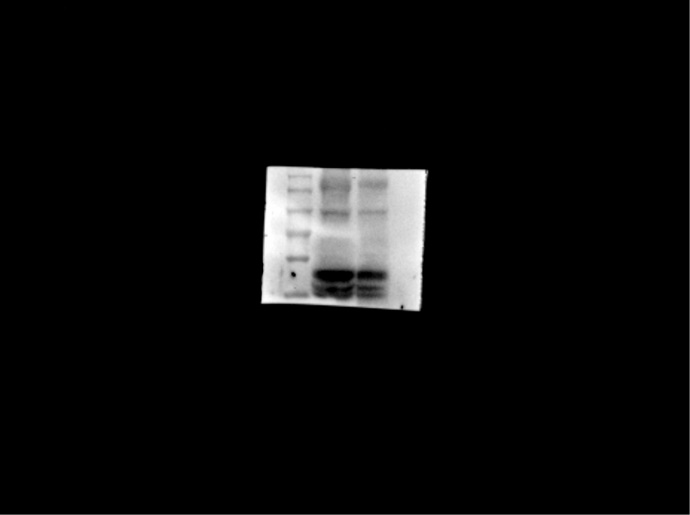


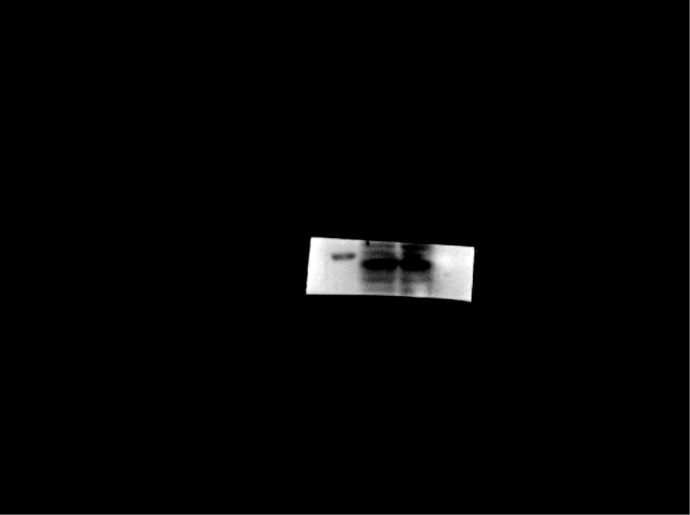
Figure 2H


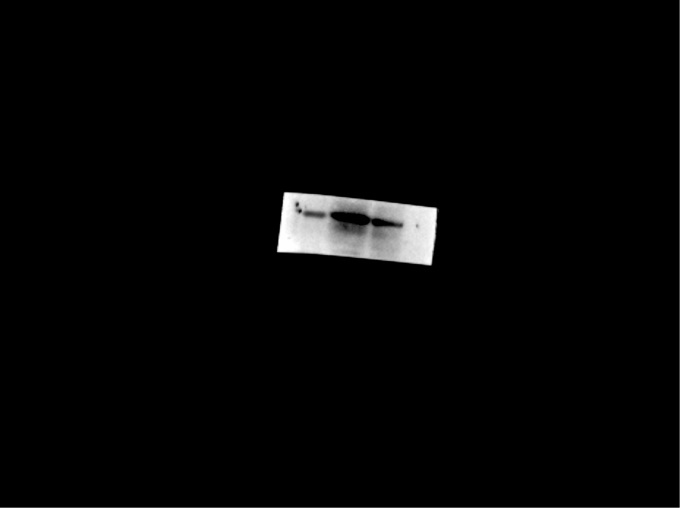


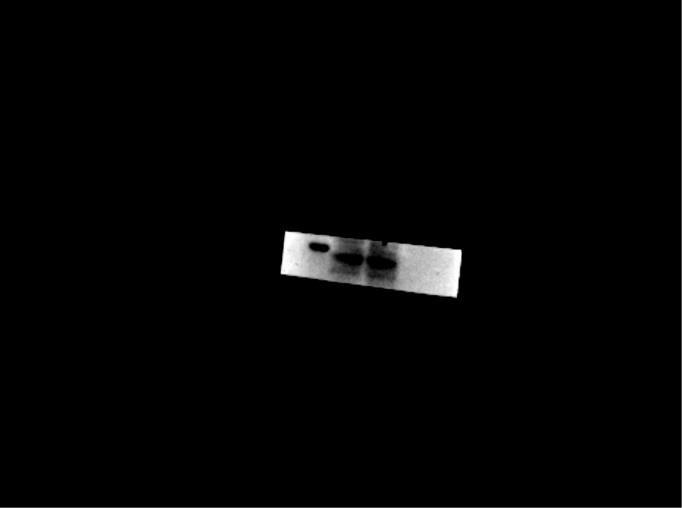


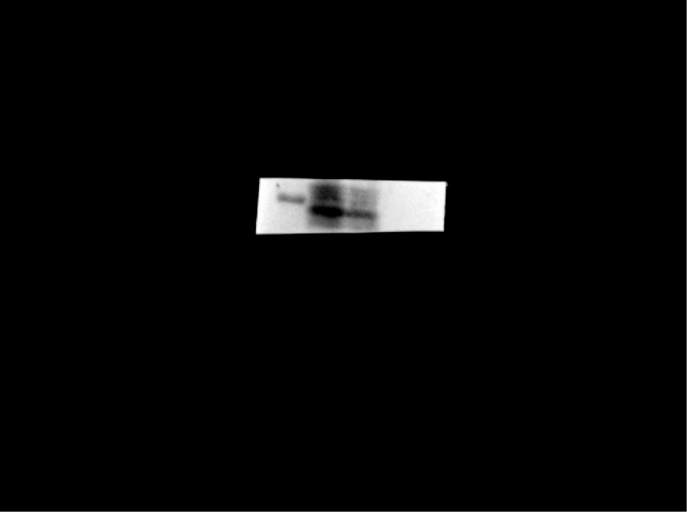


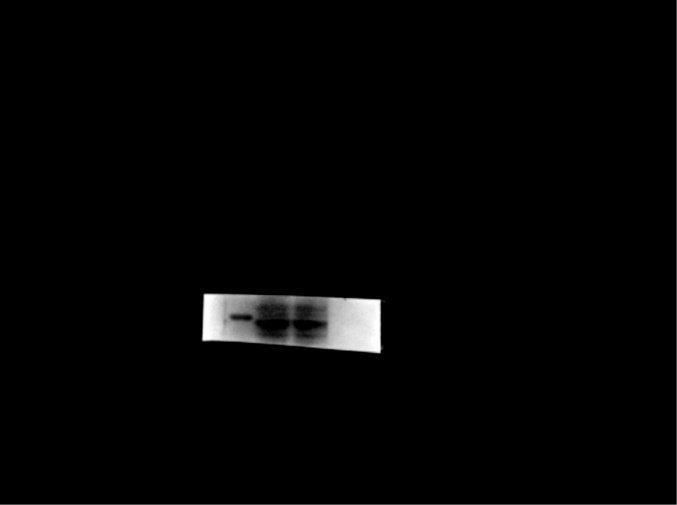


Figure 2I


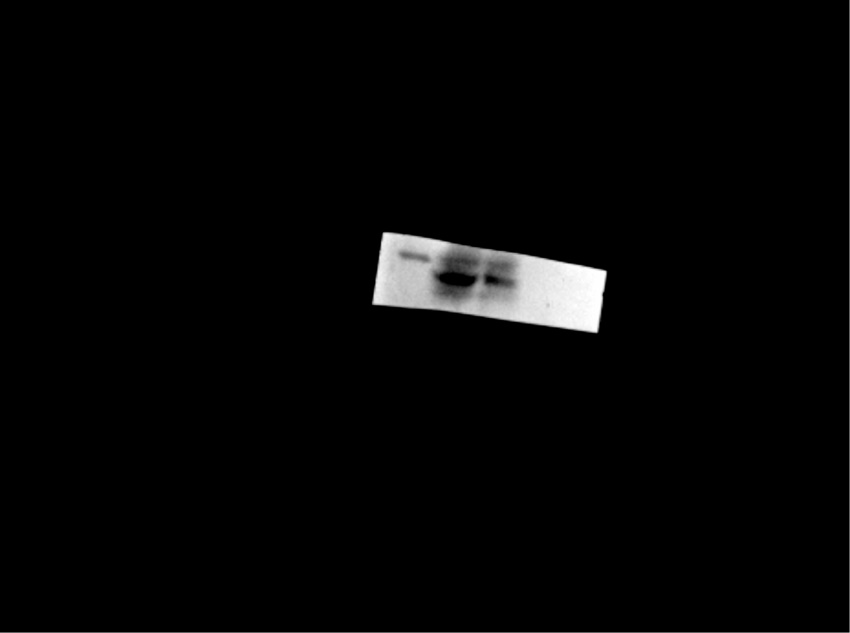


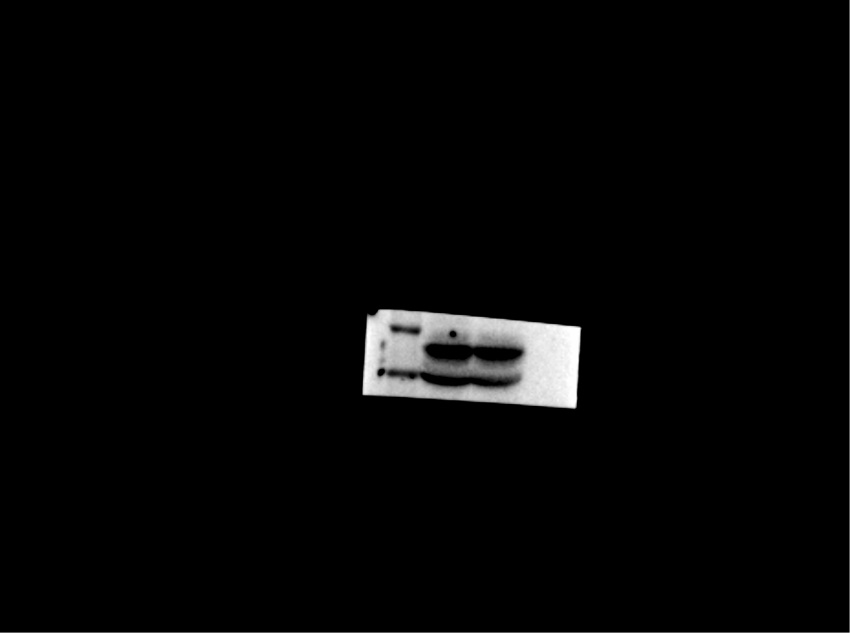


Figure 2N


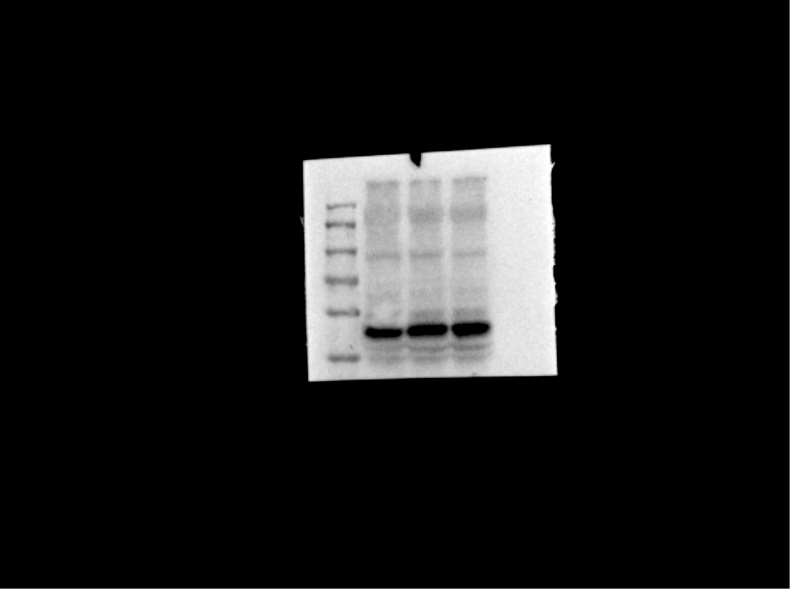


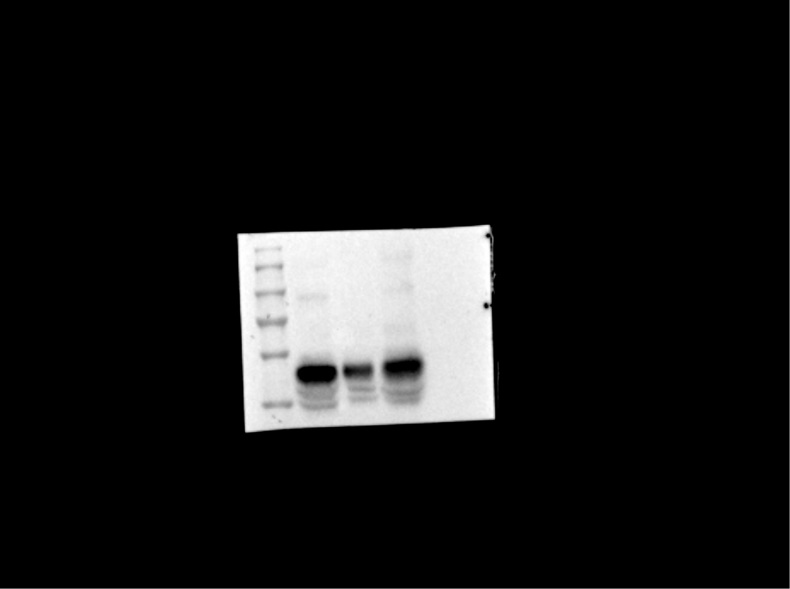


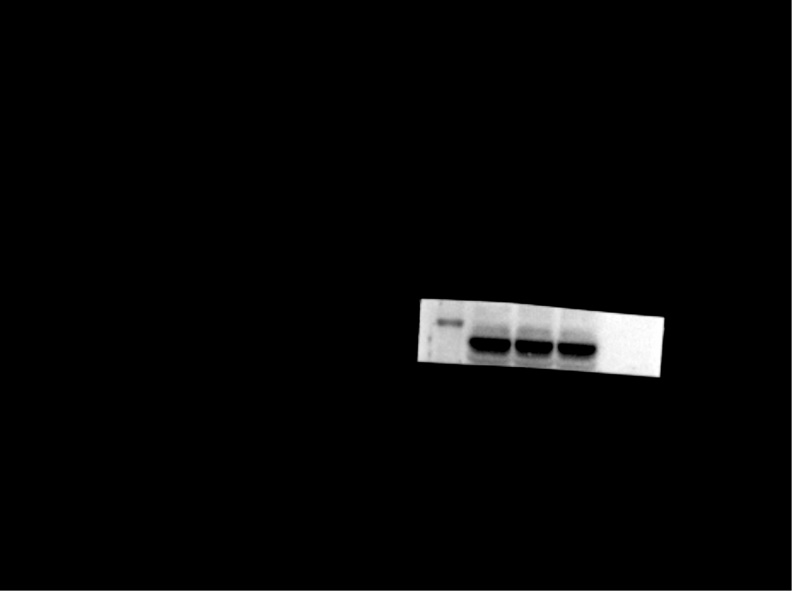


Figure 2O


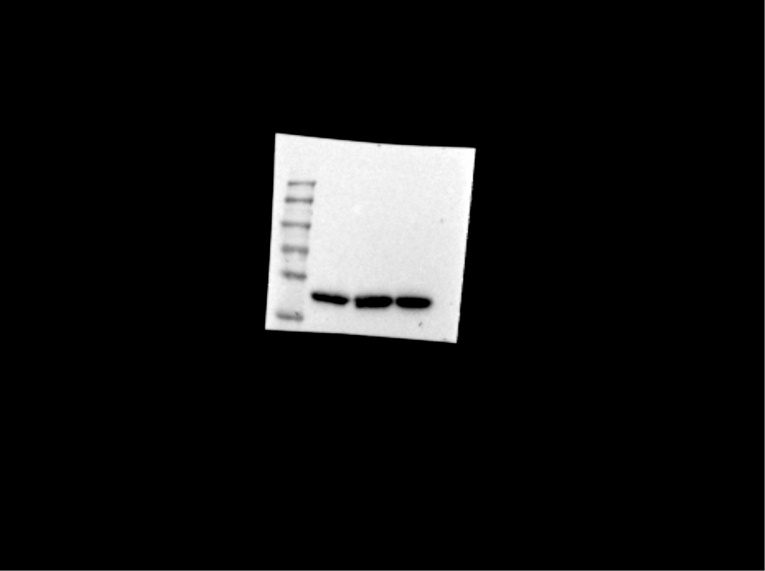


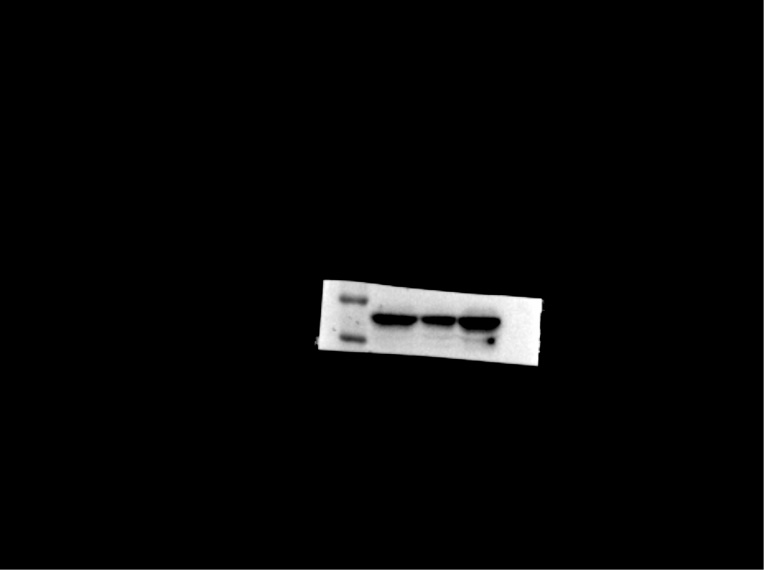


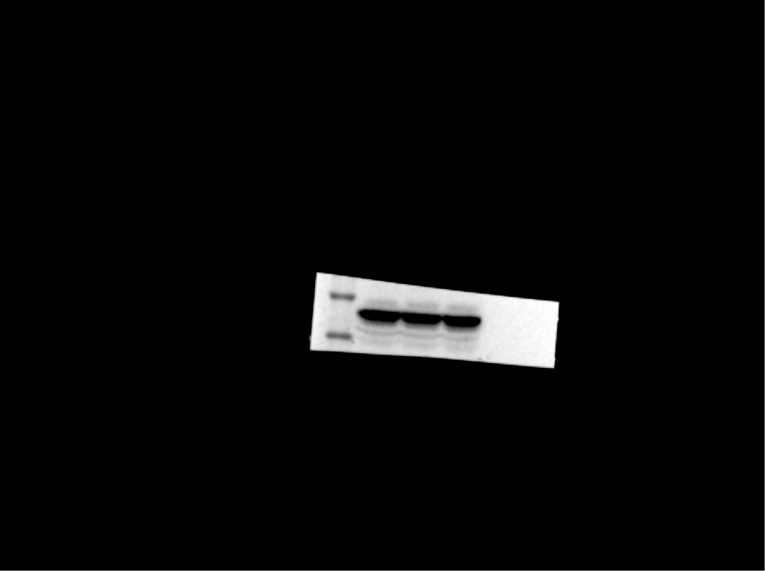


Figure 3A


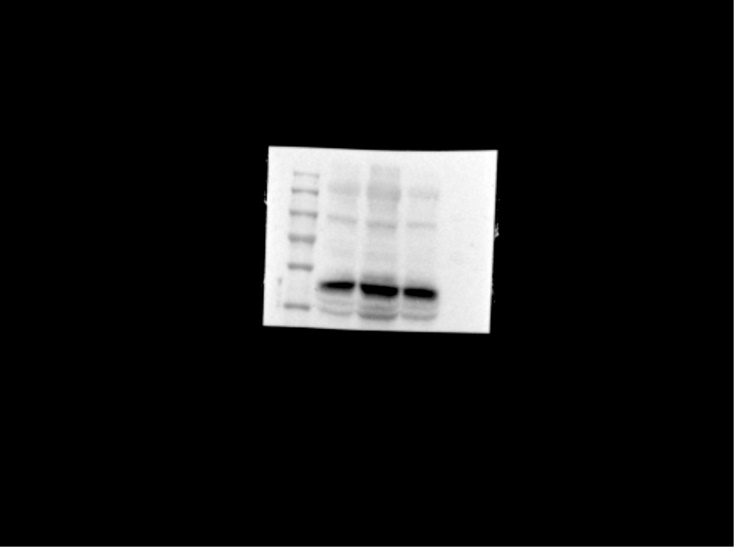


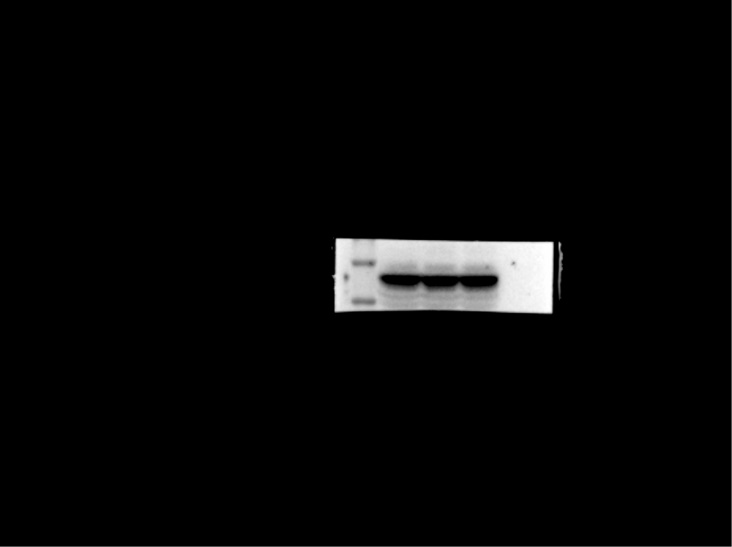


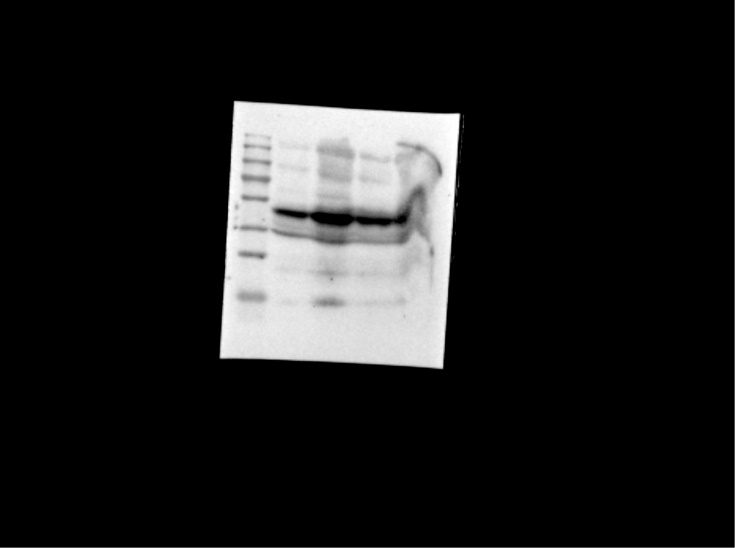


\
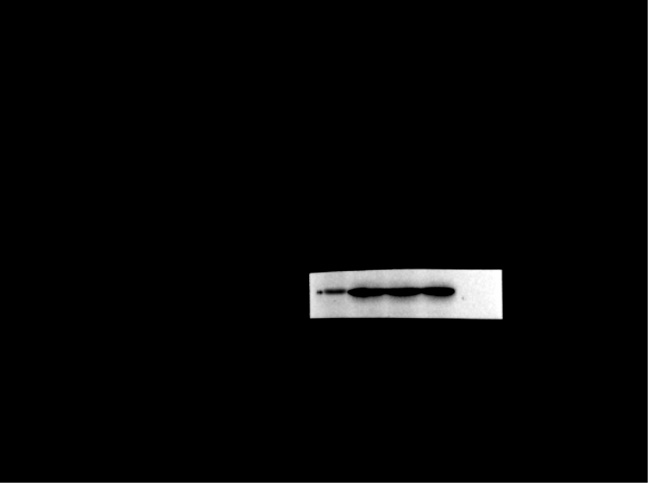
Figure 3D


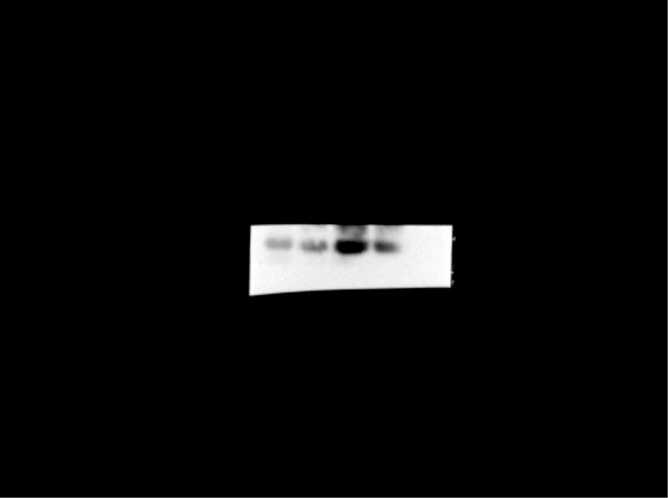


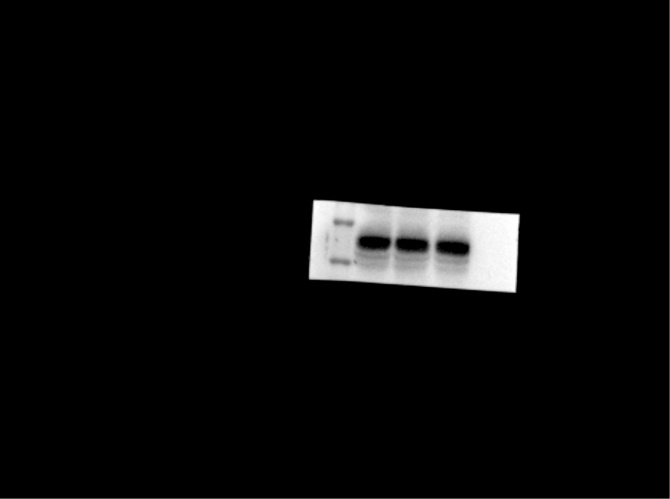


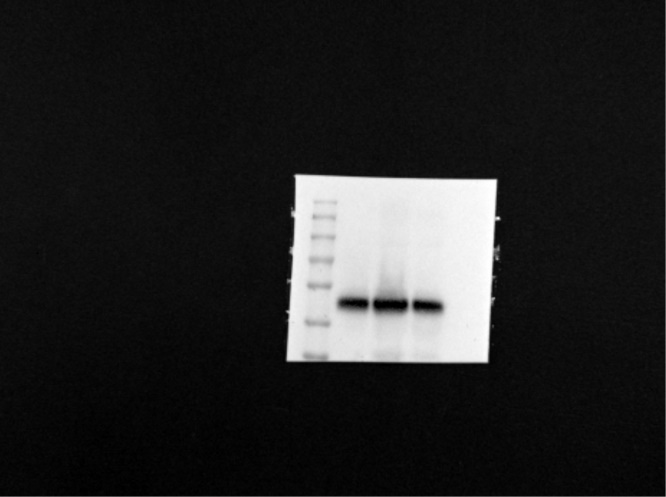


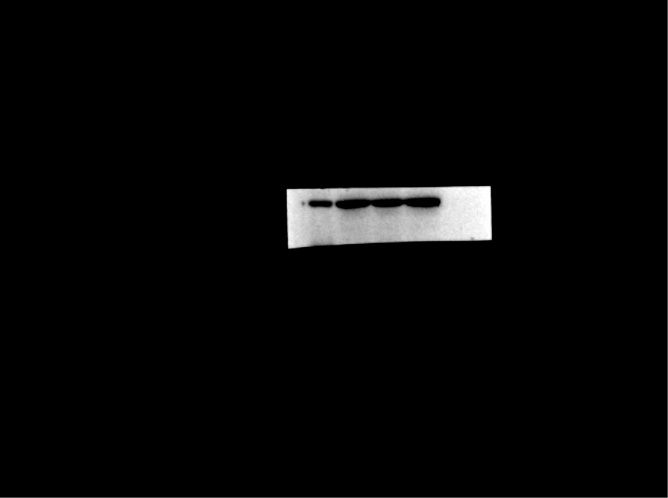


Figure 3E


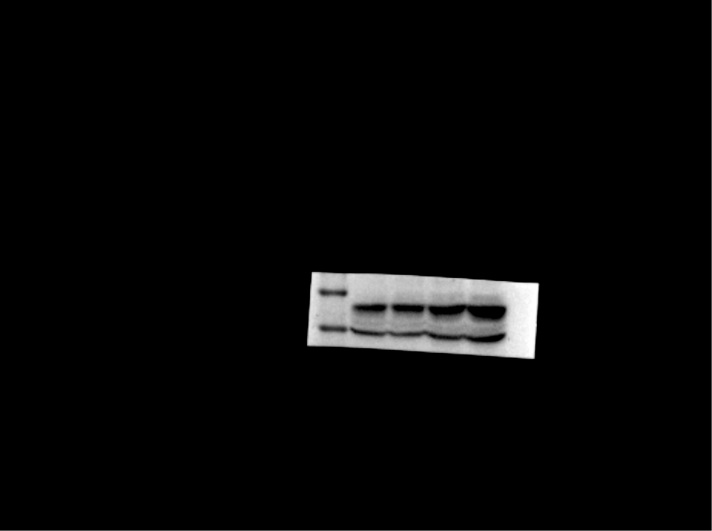


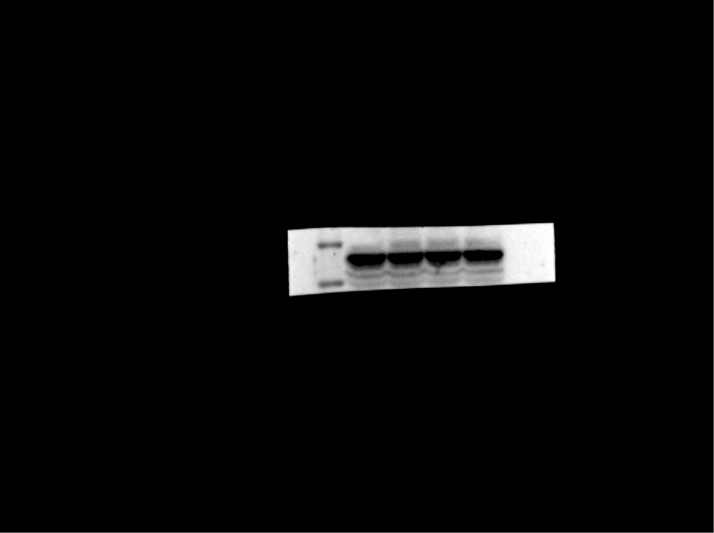


Figure 4A

Fgure
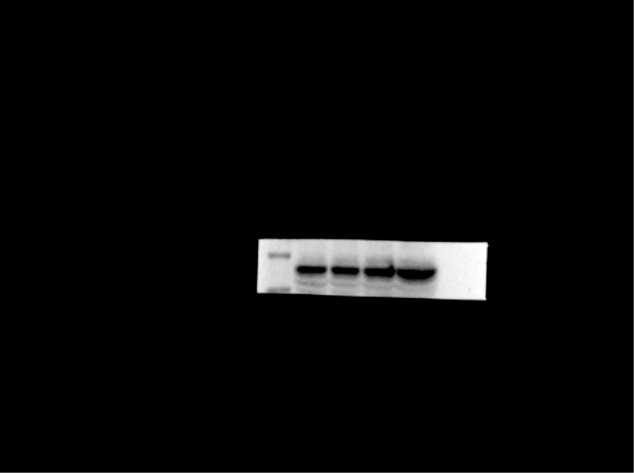


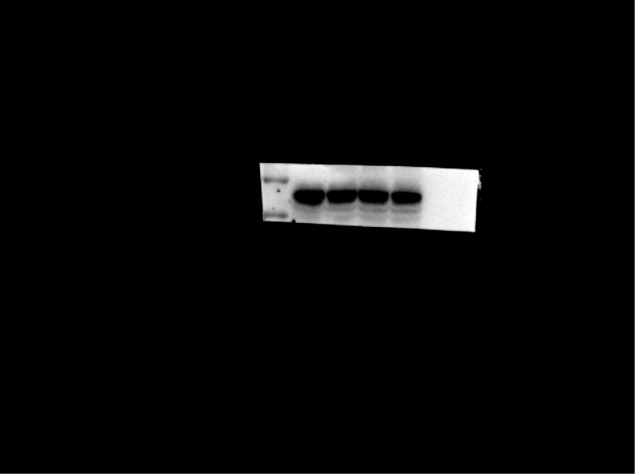


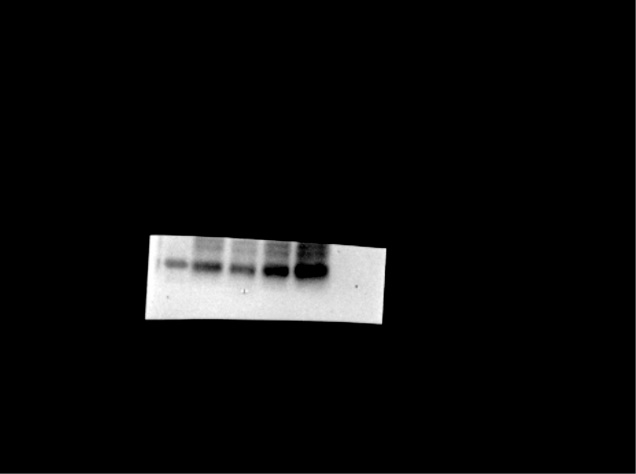


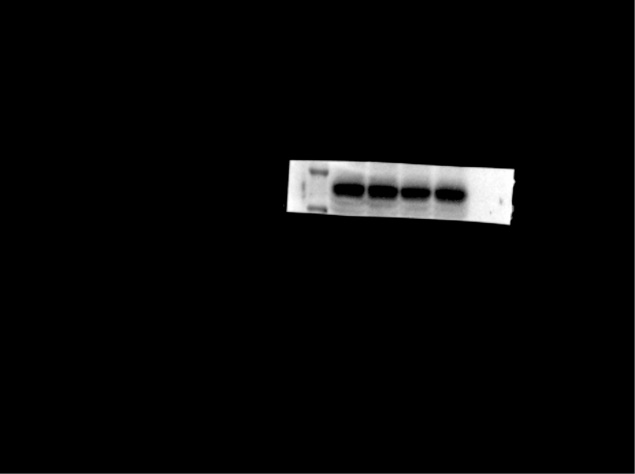


Figure 4D


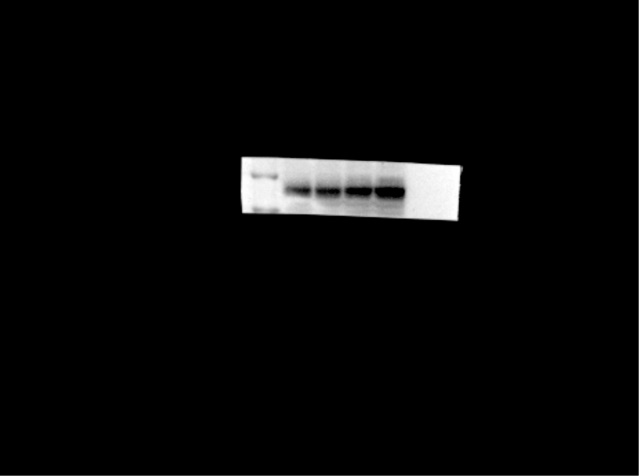


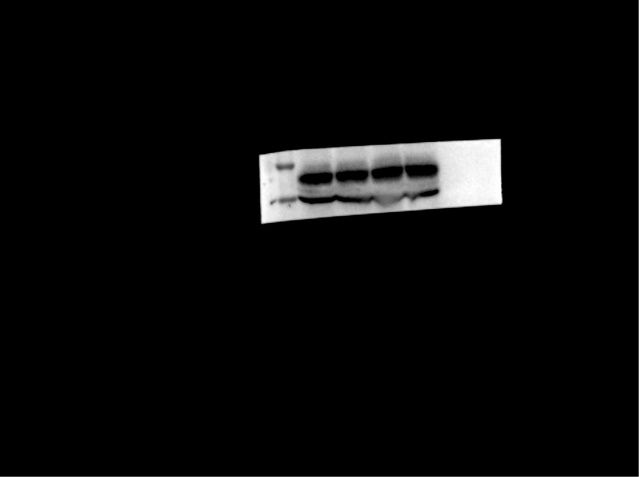


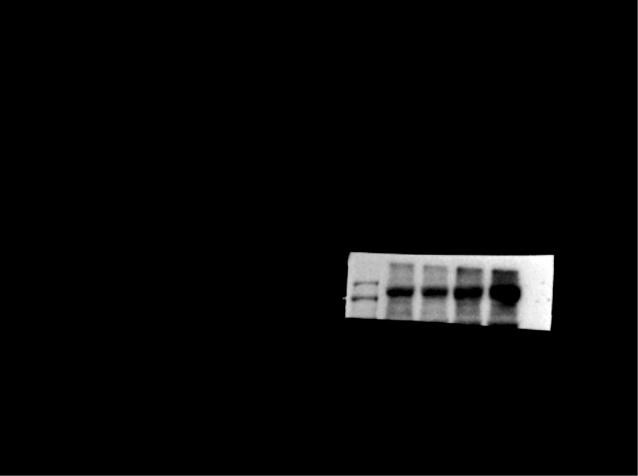


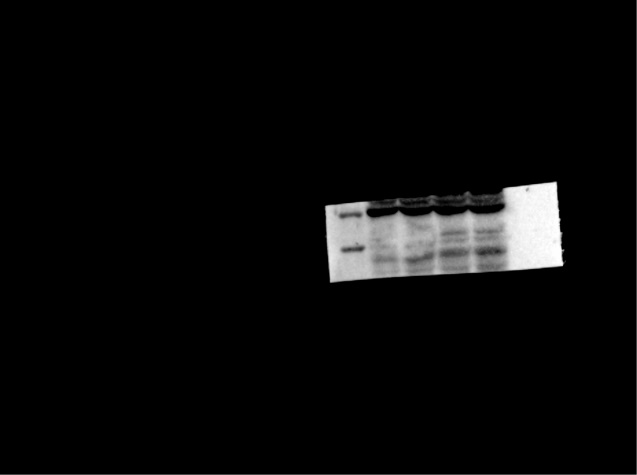


Figure 4E


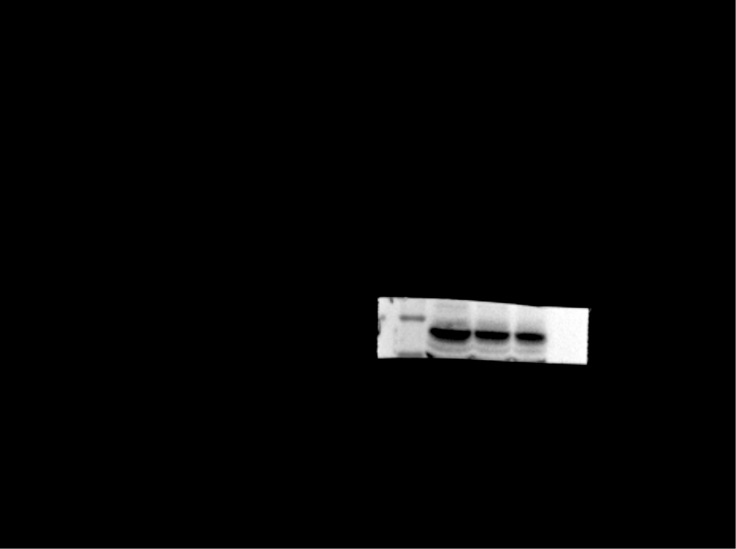


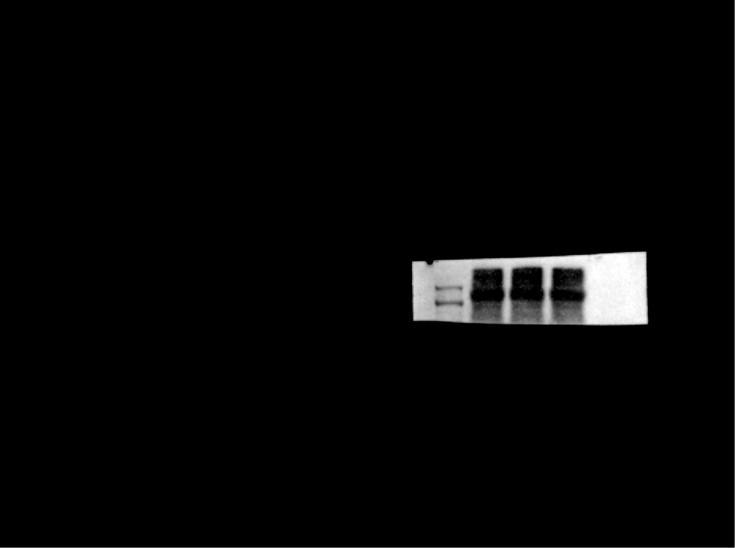


Figure S2
