# Supplementary figures and images for: ATF2-driven osteogenic activity of enoxaparin sodium-loaded polymethylmethacrylate bone cement in femoral defect regeneration
Source: J Orthop Surg Res. 2023 Aug 31;18:646. doi: 10.1186/s13018-023-04017-8 (PMC10470168; doi:10.1186/s13018-023-04017-8)

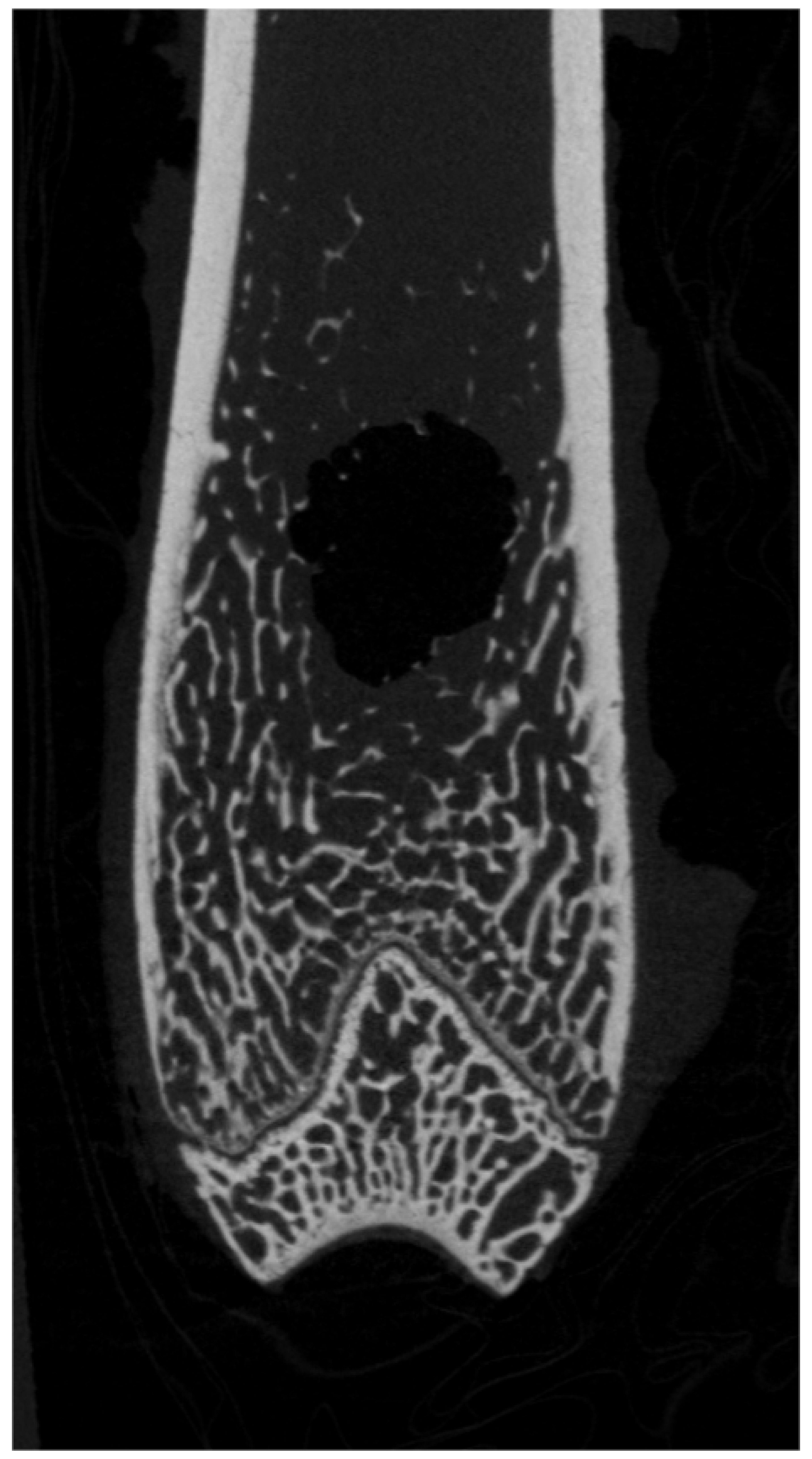

Supplement: Supplementary file 2 — Additional file 2: Figure S1 Representative micro-CT images of the femurs in rats following femoral defect models. [file 13018_2023_4017_MOESM2_ESM.jpg]

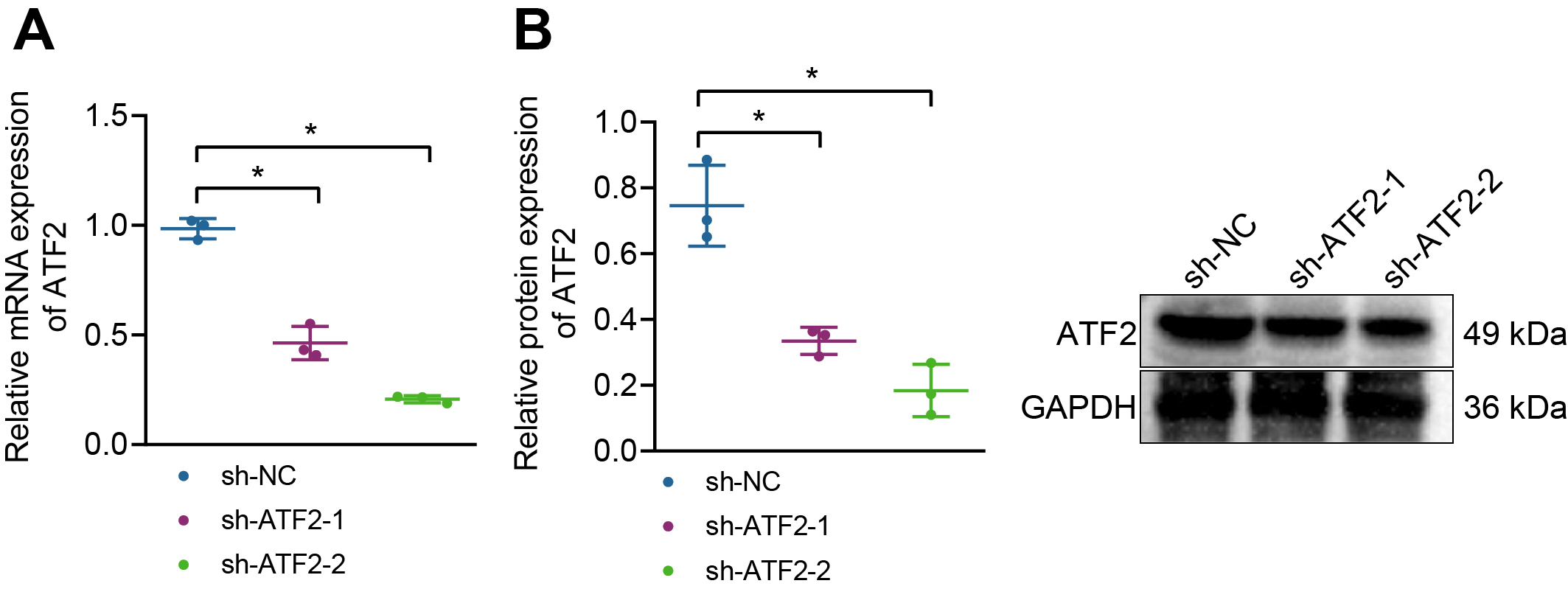

Supplement: Supplementary file 3 — Additional file 3: Figure S2 Detection of knockdown efficiency of two sh-ATF2 sequences by Western blot analysis and RT-qPCR. *p < 0.05. Cell experiments were repeated three times. [file 13018_2023_4017_MOESM3_ESM.jpg]

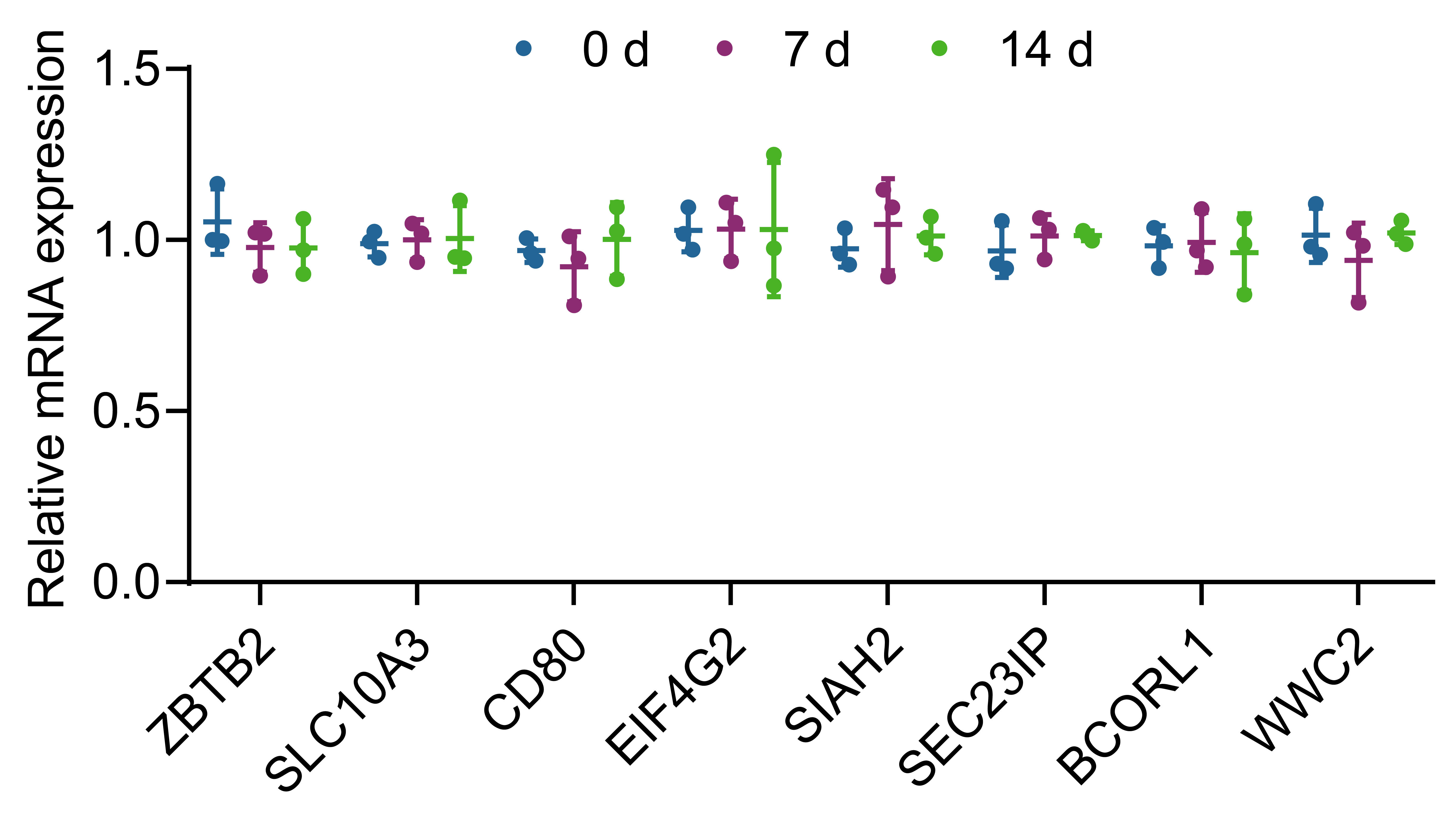

Supplement: Supplementary file 4 — Additional file 4: Figure S3 RT-qPCR detection of changes in the expression of ten target genes (ZBTB2, SLC10A3, CD80, EIF4G2, SIAH2, SEC23IP, BCORL1, and WWC2) after 0, 7, 14 days of BMSC osteogenic differentiation. Cell experiments were repeated three times. [file 13018_2023_4017_MOESM4_ESM.jpg]
