# Supplementary material for: ATF2-driven osteogenic activity of enoxaparin sodium-loaded polymethylmethacrylate bone cement in femoral defect regeneration
Source: J Orthop Surg Res. 2023 Aug 31;18:646. doi: 10.1186/s13018-023-04017-8 (PMC10470168; doi:10.1186/s13018-023-04017-8)
Supplement: Supplementary file 5 — Additional file 5: Table S1 shRNA sequences. Table S2 The qPCR primer sequences in ChIP. Table S3 RT-qPCR primer sequences [file 13018_2023_4017_MOESM5_ESM.docx]

**Table S1** shRNA sequences

| shRNA | Sequence |
| --- | --- |
| sh-NC | 5'-GTCGCGACTATAGAGTAAG-3' |
| sh-ATF2-1 | 5'-GCGAGCTAACTTGTACTTATT-3' |
| sh-ATF2-2 | 5'-ATCGTTCGTCCAGCATCATTA-3' |

Note: sh, short hairpin RNA; NC, negative control; ATF2, activating transcription factor 2;

**Table S2** The qPCR primer sequences in ChIP

| Gene | Sequence |
| --- | --- |
| miR-335-5p | Forward: 5'-ATCCAGTGCGTGTCGTG-3' |
|  | Reverse: 5'-TGCTTCAAGAGCAATAACGA-3' |

Note: qPCR, quantitative real-time PCR; ChIP, chromatin immunoprecipitation; miR-335-5p, microRNA-335-5p

**Table S3** RT-qPCR primer sequences

| Gene | Sequence |
| --- | --- |
| ATF2 | Forward: 5'-CACCAGGATAGCCCGTTACC-3' |
|  | Reverse: 5'-GCTGGACGAACGATAGCTGA-3' |
| ERK1 | Forward: 5'-GGGCCAAGCTTTTTCCCAAA-3' |
|  | Reverse: 5'-AGCCACTGGTTCATCTGTCG-3' |
| ERK2 | Forward: 5'-GGTTGTTCCCAAACGCTGAC-3' |
|  | Reverse: 5'-ATACTGCTCCAGGTACGGGT-3' |
| RUNX2 | Forward: 5'-CGCCTCACAAACAACCACAG-3' |
|  | Reverse: 5'-TCACTGCACTGAAGAGGCTG-3' |
| OCN | Forward: 5'-CGTTTAGGGCATGTGTTGCC -3' |
|  | Reverse: 5'-TGCCGTCCATACTTTCGAGG-3' |
| miR-335-5p | Forward: 5'-TCAAGAGCAATAATAACGAAAAATGT-3' |
|  | Reverse: Reverse universal primer |
| GAPDH | Forward: 5'-GCATCTTCTTGTGCAGTGCC-3' |
|  | Reverse: 5'-GATGGTGATGGGTTTCCCGT-3' |
| U6 | Forward: 5'-CGCTTCACGAATTTGCGTGTCAT -3' |
|  | Reverse: Reverse universal primer |

Note: RT-qPCR, reverse transcriptase quantitative polymerase chain reaction; ATF2, activating transcription factor 2; ERK, extracellular signal-regulated kinase; RUNX2, runt-related protein 2; miR-335-5p, microRNA-335-5p; GAPDH, glyceraldehyde-3-phosphate dehydrogenase.
